# Posttraumatic stress disorder symptom change in youth after trauma‐focused cognitive behavioral therapy: Insights from cross‐sectional and cross‐lagged panel network analysis

**DOI:** 10.1002/jts.70030

**Published:** 2025-12-24

**Authors:** Qiyue Cai, Bingyu Xu, Sydni A. J. Basha, Sun‐Kyung Lee, Stephen G. West, Abigail H. Gewirtz

**Affiliations:** ^1^ Department of Psychology Arizona State University Tempe Arizona USA; ^2^ Global Center for Applied Health Research School of Social Work Arizona State University Tempe Arizona USA; ^3^ Paul Baerwald School of Social Work & Social Welfare Hebrew University of Jerusalem Jerusalem Israel

## Abstract

Trauma‐focused cognitive behavioral therapy (TF‐CBT) is an evidence‐based treatment widely used for youth experiencing symptoms related to posttraumatic stress disorder (PTSD). This study used both cross‐sectional and cross‐lagged panel network (CLPN) analyses to examine changes in PTSD symptom networks following TF‐CBT and explore their clinical relevance. Using data from a statewide implementation of TF‐CBT, we constructed PTSD symptom networks for 652 youth (*M*_age_ = 12.47 years, 57.5% girls, 21.9% youth of color) who completed TF‐CBT and provided both pre‐ and posttreatment data. At pretreatment, central symptoms included detachment, psychological and physiological reactions, and negative cognitions. Although the overall connectivity between symptoms significantly increased after treatment, *p* = .003, the symptom structure remained stable. CLPN analyses identified symptoms with high predictive influence (out‐expected influence [out‐EI]: physiological reactions, negative emotional state, and diminished interests) and susceptibility to influence (in‐EI: internal and external avoidance, nightmare, detachment). Pretreatment centrality, *B* = 0.07, *p* < .001, and in‐EI, *B* = 0.72, *p* = .003, but not out‐EI, *B* = −0.04, *p* = .633, were associated with larger overall pre–post symptom reductions, controlling for baseline symptom severity. Improvements in symptoms with high pretreatment centrality, *B* = 1.17, *p* = .011), and in‐EI centrality, *B* = 0.34, *p* = .568, but not out‐EI centrality, *B* = 0.34, *p* = .568, were related to posttreatment psychosocial functioning over and above peripheral symptoms. These results offer preliminary evidence on how symptoms change during treatment, providing insight for understanding change mechanisms of TF‐CBT and further refining youth trauma treatment.

Trauma‐focused cognitive behavioral therapy (TF‐CBT; Cohen et al., 2006) is a well‐established, evidence‐based treatment widely used to treat youth who have experienced posttraumatic stress disorder (PTSD) symptoms after trauma exposure. TF‐CBT is a conjoint youth and parent/caregiver psychotherapy model that typically consists of 12–20 sessions. Based on needs, trained clinicians guide families through nine components, including psychoeducation, parenting skills, relaxation, affective identification and regulation, cognitive coping, trauma narrative processing, in vivo mastery, conjoint parent–child sessions, and future development. Empirical studies and meta‐analyses (Cary & McMillen, 2012; de Arellano et al., 2014; Mavranezouli et al., 2020) have demonstrated that TF‐CBT is an effective treatment in reducing PTSD symptoms among trauma‐exposed youth.

Previous research on TF‐CBT effectiveness has primarily relied on latent variable models, which assume that treatment success is a mean change in an unobserved latent construct (i.e., PTSD). In contrast, network theory argues that psychopathology arises from complex dynamic interactions among symptoms (Borsboom & Cramer, 2013; Borsboom et al., 2021). From a network perspective, psychotherapy should aim to disrupt core maladaptive symptoms and reduce their associations with other symptoms to facilitate symptom relief.

In psychopathology network analyses, *nodes* represent individual symptoms, *edges* represent the connections between symptoms, and *centrality* indices quantify the relative importance of each symptom within the network (Briganti et al., 2023). Cross‐sectional networks provide a snapshot of symptom associations at a given time point; central symptoms may serve as promising treatment targets. Temporal networks, such as the cross‐lagged panel network (CLPN) for two‐wave data, can further capture symptom stability (autoregressive effects) and directed symptom‐to‐symptom influence over time (i.e., how one symptom can influence and be influenced by other symptoms). Temporal networks may offer insight into treatment progress at the symptom level (Jordan et al., 2020). Together, these network approaches can identify important treatment processes at the symptom level, moving beyond the traditional latent variable approach (Contreras et al., 2019; Schumacher et al., 2024). However, caution is warranted: Symptom interconnections should not be equated with causal associations due to the complexity of symptom interactions and potential bidirectional associations (Bringmann et al., 2019; Rodebaugh et al., 2018).

In the field of PTSD and its treatment, both cross‐sectional and temporal network analyses have shown clinical utility. A review of 20 studies (Birkeland et al., 2020) found that cross‐sectional PTSD networks demonstrated heterogeneous centrality index rankings. However, amnesia consistently emerged as a low‐centrality symptom, and the high‐centrality symptoms typically occurred in two *Diagnostic and Statistical Manual of Mental Disorders* (5th ed., text rev.; *DSM‐5‐TR*; American Psychiatric Association [APA], 2022) symptom clusters: intrusion (Cluster B) and negative alterations in cognition and mood (NACM; Cluster D). Three treatment studies found that larger reductions in baseline high‐centrality symptoms were associated with greater overall treatment gains (Graziano et al., 2024; Papini et al., 2020; Schlesselmann et al., 2025). Longitudinal studies comparing cross‐sectional networks across time points generally show increased symptom interconnectivity following treatment (Schlesselmann et al., 2025; Xu et al., 2024).

Most studies to date have focused on adult samples; youth PTSD symptoms differ developmentally and diagnostically (APA, 2022; Scheeringa et al., 2011). Existing literature on the youth PTSD networks has mainly been observational and cross‐sectional. Results from cross‐sectional youth PTSD symptom networks have typically identified high‐centrality symptoms in *DSM‐5* Cluster B (intrusions) and/or Cluster D (NACM). Differences in centrality potentially reflect differences in measures, trauma type, developmental stage, and treatment status across studies. In their network analysis of PTSD, depression, and dysfunctional posttraumatic cognitions, de Haan et al. (2020) identified psychological and physiological reactions to trauma‐related cues and difficulty concentrating as central symptoms. Bartels et al. (2019) found that dysfunctional posttraumatic cognitions and negative emotional states were central in youth self‐reports, whereas negative cognitions, intrusive thoughts, and exaggerated startle responses were central in caregiver reports. Russell et al. (2017) found age‐related PTSD network differences after disasters. Three longitudinal observational studies (An et al., 2021; Ge et al., 2019; Liang et al., 2021) found changes in centrality indices over time, with *DSM‐5* Clusters B (intrusion) and D (NACM) remaining central. Two studies (Ge et al., 2019; Liang et al., 2021) noted increased global strength, suggesting that PTSD symptoms become more interconnected over time.

In summary, network studies of youth PTSD have identified central symptoms similar to adults (i.e., intrusion and NACM symptoms) and increased interconnectivity over time. Yet, no prior work of which we are aware has examined how symptom structure changes following evidence‐based treatment or whether centrality might be directly linked to clinical outcomes. To address this gap, the present study applied both cross‐sectional and temporal network approaches to examine PTSD symptom dynamics among trauma‐exposed youth who completed TF‐CBT. Our exploratory study had three aims. First, we aimed to describe and compare PTSD symptom networks pre‐ and posttreatment using cross‐sectional network analysis. Second, we sought to examine temporal symptom dynamics using a CLPN model. We estimated autoregressive (symptom persistence over time) and cross‐lagged effects (directed symptom‐to‐symptom influence over time) to identify symptoms with high predictive influence as well as susceptibility to influence. Finally, we explored the associations between cross‐sectional and temporal centrality and clinical outcomes.

## METHOD

### Participants

Participants were 652 youth from Minnesota, USA, who completed TF‐CBT and provided pre‐ and posttreatment PTSD data. Detailed demographic and clinical information for the sample is displayed in Table 1.

**TABLE 1:** Sample demographic and clinical background information

Variable	*M*	*SD*
Age (years)	12.47	3.12

	*n*	%
Sex		
Girl	375	57.5
Boy	267	41.0
Other	10	1.5
Race^a^		
White/European American	509	78.1
Black/African American	102	15.6
Native American	57	8.7
Latinx/Latinx American	36	5.5
Asian/Asian American	14	2.2
Hawaiian/Pacific Islander	3	0.5
Middle Eastern/North African	2	0.3
Ethnicity		
Hispanic	24	3.7
Not Hispanic	200	30.7
Unknown	31	4.8
Missing	397	60.9
Residence		
Biological parents’ home	426	65.3
Relatives/other family	87	13.3
Foster care	61	9.4
Residential care	42	6.6
Correctional facility	4	0.6
Shelter	3	0.5
Independently (alone)	2	0.3
Other or unknown	16	2.5
Missing	11	1.7
Service type		
School	125	19.2
Outpatient	86	13.2
Home	40	6.1
Residential/inpatient	30	4.6
Other	24	3.7
Missing	347	53.2
Index traumatic event		
Child maltreatment	350	53.7
Sexual assault/rape	68	10.4
Domestic violence	63	9.7
Sexual abuse	62	9.5
Physical abuse	49	7.5
Impaired caregiver	35	5.4
Emotional abuse	32	4.9
Neglect	31	4.8
Physical assault	10	1.5
Other index traumatic event	302	46.3
Bereavement	86	13.2
Separation	85	13.0
Illness/medical trauma	15	2.3
Serious accidental injury	14	2.2
Bullying	13	2.0
Attempted suicide	8	1.2
Community violence	6	0.9
Disaster	4	0.6
Witnessed suicide	4	0.6
School violence	3	0.5
Kidnapping/abduction	3	0.5
Trafficking	2	0.3
Interpersonal violence	1	0.2
Forced displacement	1	0.2
Other trauma	11	1.7
Missing	46	7.1

*Note*: *N* = 652.

^a^ Participants could select more than one.

### Procedure

This study utilized a one‐group pretest–posttest treatment study design (Shadish et al., 2002). The data were from a multiyear statewide TF‐CBT training initiative. In collaboration with the Minnesota Department of Human Services, the Ambit Network implemented 38 1‐year learning collaboratives from 2005 to 2022. A total of 1,059 providers were trained, representing diverse service settings and systems, including outpatient, inpatient/residential, child welfare, school systems, and juvenile justice. Training consisted of two 2–3‐day TF‐CBT workshops, followed by 9 months of biweekly phone consultations with clients. Providers were required to treat a minimum of five clients, gather deidentified demographic information, and conduct standardized clinical assessments at intake and follow‐up. Assessments were recommended at 3‐month intervals and upon treatment completion. The present study utilized deidentified pre‐ and posttreatment client data collected between 2015 and 2022, following the *DSM‐5‐TR* diagnostic criteria. All procedures performed in studies involving human participants were in accordance with the ethical standards of the institutional research committee at Arizona State University (STUDY00018772) and with the 1964 Helsinki Declaration and its later amendments or comparable ethical standards. The current study only used deidentified data collected for clinical training purposes. Parents provided consent for their children to receive treatment.

### Measures

#### Posttraumatic stress symptoms

Clinical interviews were conducted to assess youth posttraumatic stress symptoms (PTSS) using the 31‐item UCLA PTSD Reaction Index for *DSM*‐5 (PTSD‐RI‐5; Pynoos & Steinberg, 2015). The PTSD‐RI‐5 assesses symptoms from each *DSM‐5* symptom cluster, including five intrusion items (Cluster B), two avoidance items (Cluster C), 13 NACM items (Cluster D), and seven hyperarousal items (Cluster E), with the remaining four items screening for the dissociative subtype of PTSD. Respondents were asked to rate symptom frequency on a 5‐point Likert scale ranging from 0 (*none of the time*) to 4 (*most of the time*), with scores summed and higher scores indicating higher PTSS levels. The measure has demonstrated good psychometric properties in previous validation studies (e.g., Cronbach's α = .94; Kaplow et al., 2020). In the current sample, the PTSD‐RI‐5 exhibited excellent internal consistency at both pretreatment, Cronbach's α = .90, and posttreatment, Cronbach's α = .91.

#### Psychosocial difficulties

Youth psychosocial difficulties were measured using the Strengths and Difficulties Questionnaire (SDQ; Goodman, 2001; coefficient α = .73), a 25‐item measure that assesses youth psychosocial functioning over the last 6 months that has demonstrated sound psychometric properties (e.g., Cronbach's α = .73). Items are rated on a 3‐point scale ranging from 0 (*not true*) to 2 (*certainly true*), with a Total Difficulties score calculated by summing scores across the Emotional Problems, Conduct Problems, Hyperactivity/Inattention, and Peer Relationship Problems subscales. Although clinicians attempted to obtain the SDQ from parents, youth, and teachers, we only included parent‐report in the current analyses, as it represents the largest sample size and, in this study, used a different reporter than the measure of PTSS. In the current sample, the SDQ Total Difficulties score exhibited adequate internal consistency at both pretreatment, Cronbach's α = .68, and posttreatment, Cronbach's α = .65.

#### Covariates

Youth age at pretreatment (in years), sex (0 = boy, 1 = girl), racial minority status (0 = white, 1 = racial minority), and index traumatic event were included as covariates. We categorized index trauma into two groups given the diverse trauma indices reported (Table 1): child maltreatment (e.g., physical abuse, sexual abuse, neglect) and other index trauma (e.g., accidents, natural disasters, community violence).

### Data analysis

Network analysis followed standard procedures (Epskamp et al., 2018) and reporting guidelines (Burger et al., 2023). All analyses were conducted in R (Version 4.3.3; R Core Team, 2024).

#### Cross‐sectional networks and comparison

##### Model estimation

We conducted cross‐sectional, unidirectional PTSD symptom networks at pre‐ and posttreatment using Gaussian graphical models (GGMs) with graphical least absolute shrinkage and selection operation (LASSO) regularization. The model used the extended Bayesian information criterion (EBICglasso) with a gamma (γ) hyperparameter set to .5 (Isvoranu & Epskamp, 2023). This procedure retains partial correlations with the largest magnitude and removes partial correlations of minimal magnitude that are likely spurious. The *estimateNetwork* function within the *bootnet* package in R (Epskamp et al., 2018) was used.

##### Model visualization

Networks were visualized using the *qgraph* package (Epskamp et al., 2012) with the *spring* layout. Nodes positioned closer together are more strongly connected, edge thickness reflects partial correlation magnitude, and edge color (blue vs. red) indicates positive versus negative associations.

##### Centrality estimation

Expected influence (EI) was used to quantify node centrality. EI sums the weights of all edges connected to a node while preserving their sign, reflecting a node's overall impact on the network (Borsboom et al., 2021). We chose EI over strength because EI is more interpretable with directional and negative connections. EI is functionally equivalent to strength when all edges are positive. In the current study, central symptoms were defined as having EI *z* scores greater than 1.0.

##### Stability and accuracy

Network stability and accuracy were evaluated through nonparametric bootstrap procedures with 1,000 bootstrap samples using the *bootnet* package. Edge weight accuracy was evaluated by bootstrapped confidence intervals (CI). We computed the correlation stability coefficient (CS‐coefficient) for centrality via the case‐dropping bootstrap. The CS‐coefficient is roughly categorized into unstable (< .25), moderate (.25–.5), and preferred (> .50; Epskamp et al., 2018). Bootstrapped difference tests were conducted to identify significant differences between edges and centrality within the same network.

##### Network comparison

To assess whether network characteristics differed from pre‐ to posttreatment, we used the *NCT* function within the *NetworkComparsionTest* package with 1,000 permutations (van Borkulo et al., 2023). Because the same participants were assessed at both time points, paired network comparison tests (NCT) were performed to examine potential differences in network structure and global strength. Bonferroni correction was applied to adjust for multiple comparisons.

##### Subgroup analysis

We conducted subgroup analyses across key demographic variables (i.e., age, sex, race, and index trauma). For each subgroup, we estimated separate pre‐ and posttreatment cross‐sectional networks, computed EI, and tested within‐group pre–post differences using the paired NCT. Additionally, we performed between‐group NCTs at each time point (e.g., boys vs. girls at pretreatment) to identify potential effect modifiers that would restrict generalization (Degtiar & Rose, 2023); these results are shown in Supplementary S4.

#### CLPNs

##### Model estimation

We initially conducted CLPNs without any covariates to model the temporal dynamics between PTSD symptoms from pretreatment to posttreatment, using the *estimateNetwork* function within the *bootnet* package. We used LASSO regularization with 10‐fold cross‐validation to shrink small regression coefficients to 0 and prevent overfitting (Funkhouser et al., 2021; Williams et al., 2019).

##### Model visualization

We visualized the standardized autoregressive and cross‐lagged effects separately. Directed arrows were used to indicate the direction from pretreatment to posttreatment in the cross‐lagged plots. To improve interpretability, cross‐lagged effects with an absolute value greater than 0.10 are displayed.

For centrality estimation, we computed EI for both out‐degree EI (out‐EI; the degree to which a symptom predicted other symptoms) and in‐degree EI (in‐EI; the degree to which a symptom was predicted by other symptoms). Similar to the cross‐sectional networks, we assessed stability and accuracy by reporting edge‐weight accuracy, CS‐coefficients, and bootstrapped difference tests through nonparametric bootstrapping procedures with 1,000 bootstrap replications. To probe the robustness and generalizability of the CLPN results, we conducted a sensitivity analysis by estimating a second model that statistically controlled for demographic covariates (age, sex, race, and index trauma). This analysis allowed us to examine whether CLPN remains stable when accounting for potential confounders.

#### Symptom centrality and clinical outcomes

To assess whether symptom centrality was associated with clinical outcomes, we conducted both symptom‐level analyses and separate outcome analyses with broader psychosocial difficulties using both pretreatment and temporal centrality indices as predictors.

##### Symptom‐level analysis

Following Graziano et al. (2024) and Schlesselmann et al. (2025), we first examined whether pretreatment centrality (EI) was associated with symptom importance in the broader pre–post symptom change process. For each symptom, we first computed change scores (post–pre) and then calculated the Pearson correlation between a symptom's change score and the change scores of the remaining 19 symptoms. This correlation indexed the extent to which the change in a given symptom co‐occurred with broader symptom change, or each symptom's importance in the broader pre–post change process. We then tested whether this index was related to pretreatment EI by regressing the correlation coefficient on EI, controlling for baseline severity. Extending this method to the CLPN, we further examined whether a symptom's importance in the broader symptom change processes was related to CLPN in‐EI and out‐EI.

##### Broader psychosocial outcome

We calculated the average change score for all central symptoms identified in the pretreatment network (i.e., EI *z* score >1) as well as for the average change score for the remaining peripheral symptoms. A linear regression was conducted to examine whether changes in central PTSD symptoms were associated with youth psychosocial difficulties over and above changes in peripheral PTSD symptoms, while controlling for baseline psychosocial difficulties, baseline PTSD symptoms, and selected covariates (age, sex, and racial minority status; Cohen et al., 2003). Similarly, we identified central symptoms based on the CLPN (in‐EI or out‐EI *z*‐score >1) and conducted a parallel regression analysis.

#### Missing data

For network construction, we used listwise deletion, including only participants with both pre‐ and posttreatment data. Participants with sparse or missing data at either time point were excluded, as multiple imputation would lead to minimal adjustment and very large standard errors in the network analysis (Enders, 2022). The regression analysis included another clinical outcome (SDQ) and covariates. Data were missing primarily due to a failure to consistently collect parent‐report SDQ at both time points. Little's (1988) missing completely at random assumption was not met, χ^2^(17, *N* = 652) = 136.19, *p* < .001. Full information maximum likelihood, which addresses data that are missing at random (Enders, 2022), was used to handle missing data using the *lavaan* package (Rosseel et al., 2014).

## RESULTS

### Descriptive results

Supplementary Table S1 summarizes the demographic and clinical measures at pretreatment. Youth reported a mean total PTSS score of 36.4 (*SD* = 16.33), higher than the recommended cutoff score of 35 for a probable PTSD diagnosis (Kaplow et al., 2020). At posttreatment, youth showed significant improvement in PTSS, *d* = 0.99, and psychosocial difficulties, *d* = 0.33.

### Cross‐sectional networks and comparison

#### Pretreatment PTSD network

**FIGURE 1:**
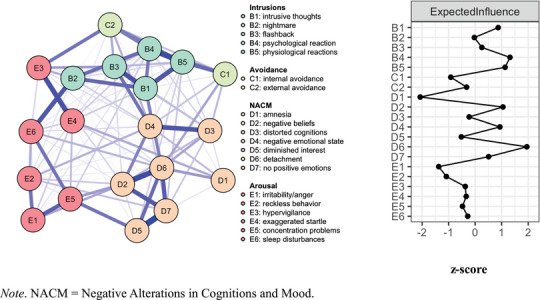
Pretreatment (A) posttraumatic stress disorder symptom network and (B) expected influence (EI) values *Note*: EI values (Panel B) are depicted as *z* scores. NACM = negative alterations in cognitions and mood.

The pretreatment PTSD network (Figure 1, Panel A) indicated strong positive cross‐sectional connections between apathy (D5) and restricted affect (D7); psychological reactions (B4) and physiological reactions (B5); and negative cognitions (D2) and detachment (D6). Bootstrapped edge weight estimates closely aligned with the original sample estimates across the network, with an average 95% confidence interval (CI) width of 0.12 (*SD* = 0.05; Supplementary Figure S1, Panel A; Supplementary Table S2). Bootstrapped edge‐weight difference tests are shown in Supplementary Figure S2, Panel A.

The most central symptoms based on EI (Figure 1, Panel B; Supplementary Table S3) included detachment (D6), psychological reactions (B4), physiological reactions (B5), and negative cognitions (D2). CS‐coefficients indicated strong EI centrality stability, CS(cor = 0.7) = 0.75. Bootstrapped centrality difference tests (Supplementary Figure S3, Panel A; Supplementary Figure S4, Panel A) further supported that detachment (D6) was significantly more central than 14 other symptoms, whereas amnesia (D1) was significantly less central than 18 other symptoms.

#### Posttreatment PTSD network

**FIGURE 2:**
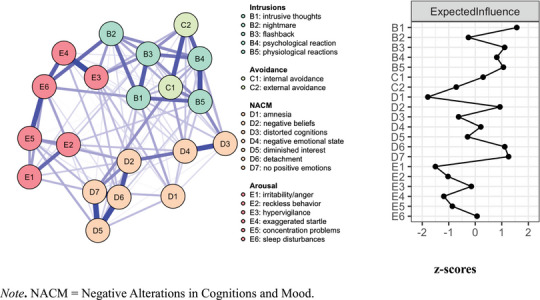
Posttreatment (A) posttraumatic stress disorder symptom network and (B) expected influence (EI) values *Note*: EI values (Panel B) are depicted as *z* scores. NACM = negative alterations in cognitions and mood.

The posttreatment network (Figure 2, Panel A) closely resembled the pretreatment network structure, with the same strong positive connected pairs. Bootstrapped edge weight estimates closely aligned with the sample estimates, with an average 95% confidence interval width of 0.13 (*SD* = 0.06; Supplementary Figure S1, Panel B; Supplementary Table S2). Bootstrapped edge weight difference tests are shown in Supplementary Figure S2 (Panel B).

The most central symptoms based on EI (Figure 2, Panel B; Supplementary Table S3) included intrusive thoughts (B1), restricted affect (D7), detachment (D6), flashbacks (B3), and physiological reactions (B5). CS‐coefficients indicated strong EI centrality stability, CS(cor = 0.7) = 0.75 (Supplementary Figure S3, Panel B). Bootstrapped centrality difference tests (Supplementary Figure S4, Panel B) supported that intrusive thoughts (B1) were significantly more central than 13 other symptoms, whereas amnesia (D1) was significantly less central than 16 other symptoms.

#### Network comparison test

The results from the paired NCT showed no significant difference in network structure between the pre‐ and posttreatment PTSD networks (*M* = 0.11), *p* = .915. In contrast, global network strength significantly increased, *S* = 0.49, *p* = .003. At the within‐person level, the average variability across PTSD symptoms was significantly higher at pretreatment (*M* = 1.23, *SD* = 0.29) than posttreatment (*M* = 0.93, *SD* = 0.37), paired *t*(651) = 17.48, *p* < .001. The results of sensitivity analyses with NCT results are shown in Supplementary Table S4.

### Temporal networks

#### Unadjusted CLPN

**FIGURE 3:**
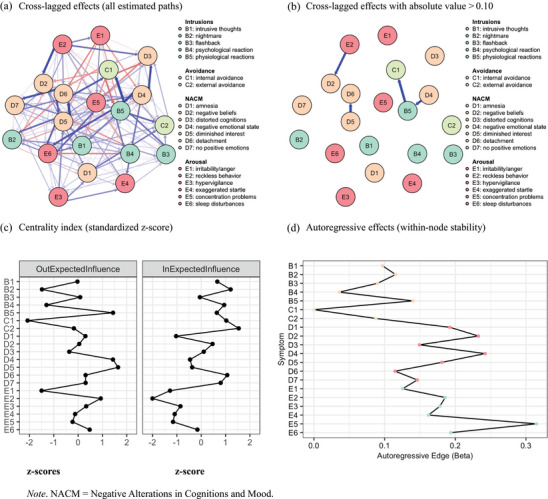
Cross‐lagged panel network analysis of the posttraumatic stress disorder (PTSD) symptom network and centrality indices *Note*: The figure depicts (A) standardized cross‐lagged effects with all estimated paths, (B) standardized cross‐lagged effects with strong estimated paths (absolute value > 0.10), (C) symptom centrality indices (*z* scores), and (D) standardized autoregressive effects. NACM = negative alterations in cognitions and mood.

Figure 3 presents all estimated CLPN cross‐lagged effects without covariates (Panel A) and displays the stronger directional associations (>.10) between pre‐ and posttreatment symptoms (Panel B). The strongest cross‐lagged edges were observed between diminished interest (D5) and detachment (D6), reckless behavior (E2) and negative beliefs (D2), and negative emotional state (D4), physiological reactions (B5), and internal avoidance (C1). Bootstrapped edge weight estimates aligned moderately with the sample estimates, with an average 95% confidence interval width of 0.11 (*SD* = 0.03; Supplementary Figure S1, Panel C; Supplementary Table S2). Bootstrapped edge weight difference tests are shown in Supplementary Figure S2 (Panel C).

Centrality estimates for the CLPN are shown in Figure 3 (Panel C) and Supplementary Table S2. Symptoms with the highest out‐EI included physiological reactions (B5), negative emotional state (D4), and diminished interest (D5). Symptoms with the highest in‐EI included external avoidance (C2), nightmares (B2), detachment (D6), and internal avoidance (C1). The correlation stability coefficients for EI were low‐to‐moderate: out‐EI CS(cor = .7) = .28, in‐EI CS(cor = .7) = .36 (Supplementary Figure S3, Panel C). To evaluate whether the number of cross‐validation folds influenced stability, we reran the CLPN with 5‐fold cross‐validation, yielding a consistent network structure with low‐to‐moderate stability, out‐EI CS(cor = .7) = .28, in‐EI CS(cor = 0.7) = .28. As these values fall below the preferred threshold of .50 (Epskamp et al., 2018), they should be interpreted with caution. Bootstrapped centrality difference tests for both in‐EI and out‐EI are provided in Supplementary Figure S4.

The average standardized autoregressive coefficient (Figure 3, Panel D; Supplementary Table S5) was estimated to be .15 (*SD* = 0.07), substantially stronger than the average standardized cross‐lagged coefficient value, β = .01 (*SD* = 0.01), *t*(19) = 8.49. *p* < .001. The strongest autoregressive effects were found for concentration problems (E5), negative emotional states (D4), and negative beliefs (D2). In contrast, the lowest autoregressive coefficients were found for internal avoidance (C1) and psychological reactions (B4).

#### Covariate‐adjusted CLPN

We reestimated CLPN after adjusting for age, sex, race, and index trauma. The resulting network remained largely consistent with the unadjusted model in terms of overall structure, central symptoms, and the strongest autoregressive and cross‐lagged paths. Visualizations of the covariate‐adjusted CLPN are presented in Supplementary Figure S5 and Supplementary Table S3. Stability and accuracy indices are presented in Panel D of Supplementary Figures S1, S2, and S3 and Panels E–F of Supplementary Figure S4.

### Centrality and clinical outcomes

**FIGURE 4:**
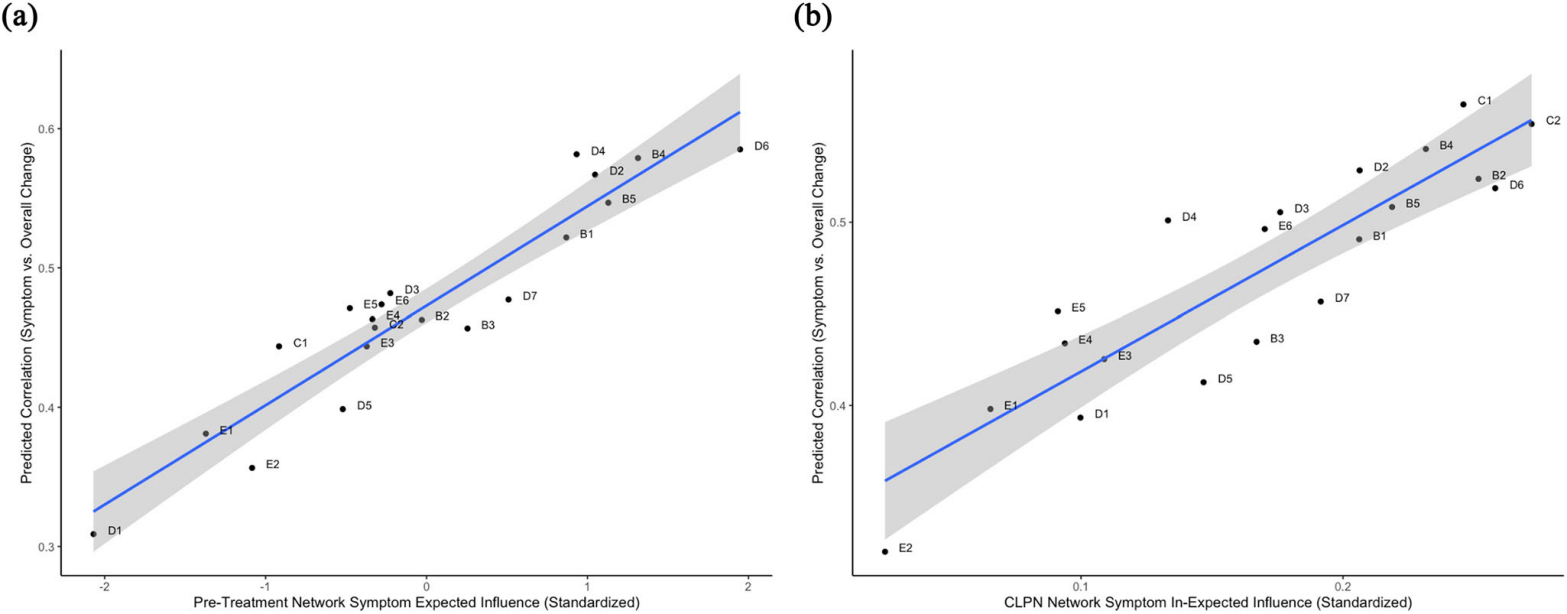
Scatterplot of (A) expected influence from the pretreatment network and (B) in‐degree expected influence versus the predicted correlation of each symptom's change with changes in the remaining 19 symptoms *Note*: Solid lines indicate the regression fit, and gray shaded bands represent the 95% confidence interval (CI). CLPN = cross‐lagged panel network.

Baseline symptom severity was not related to pretreatment EI, *r* = 0.2, *p* = .400; CLPN in‐EI, *r* = .2, *p* = .408; or CLPN out‐EI, *r* = −.29, *p* = .211. Linear regression (Figure 4, Panel A) showed that pretreatment EI was significantly associated with the correlation between change in a given symptom and changes in other symptoms, controlling for baseline severity, *B* = 0.07 (*SE* = 0.01), *p* < .001. When controlling for baseline severity, in‐EI (Figure 4, Panel B), *B* = 0.72 (*SE* = 0.20), *p* = .003, but not out‐EI, *B* = −0.04 (*SE* = 0.08), *p* = .633, was significantly related to a symptom's importance in the broader pre–post change process.

As shown in Table 2, improvements in central PTSD symptoms identified in the pretreatment network were associated with better posttreatment psychosocial functioning over and above changes in peripheral symptoms, *B* = 1.17 (*SE* = 0.46), *p* = .011. Improvements in central symptoms identified from CLPN in‐EI, *B* = 0.34 (*SE* = 0.59), *p* = .568, were significantly related to better psychosocial functioning over and above changes in peripheral symptoms.

**TABLE 2:** Linear regression analysis predicting posttreatment Strengths and Difficulties Questionnaire (SDQ) total scores

Variable	*B*	*SE*	*t*(652)	*p*
Pretreatment network centrality and SDQ				
Baseline SDQ total	0.63	0.05	11.98	< .001
Baseline PTSD‐RI‐5	0.10	0.03	3.38	.001
Age	−0.31	0.12	−2.62	.009
Sex^a^	−0.28	0.72	−0.38	.701
Racial minority^a^	0.07	0.90	0.08	.937
Child maltreatment^a^	−0.37	0.70	−0.53	.594
Other symptom change	0.57	0.68	0.84	.401
Core symptom change (EI)	1.17	0.46	2.54	.011
CLPN network centrality and SDQ				
Baseline SDQ total	0.64	0.05	12.22	< .001
Baseline PTSD‐RI‐5	0.10	0.03	3.27	.001
Age	−0.31	0.12	−2.55	.011
Sex^a^	−0.33	0.73	−0.45	.650
Racial minority^a^	0.20	0.90	0.22	.826
Child maltreatment^a^	−0.27	0.70	−0.38	.702
Other symptom change	0.48	0.71	0.67	.502
Core symptom change (in‐EI)	1.07	0.49	2.21	.027
Core symptom change (out‐EI)	0.34	0.59	0.57	.568

*Note*: CLPN = cross‐lagged panel network; PTSD‐RI‐5 = UCLA PTSD Reaction Index for *DSM‐5*; EI = expected influence; in‐EI = in‐degree EI; out‐EI = out‐degree EI.

^a^ Sex: 1 = girl, 0 = boy; racial minority: 1 = racial minority, 0 = White; childhood maltreatment: 1 = childhood maltreatment index trauma, 0 = other index trauma.

## DISCUSSION

The study described and compared cross‐sectional PTSD symptom networks at pre‐ and posttreatment. Although core symptoms varied slightly across demographic subgroups, all high‐centrality symptoms emerged from *DSM‐5* Clusters B (intrusion) and D (NACM), consistent with prior research in both adult and youth samples (Bartels et al., 2019; Birkeland et al., 2020; de Haan et al., 2020; Ge et al., 2019; Pfeiffer et al., 2019). Thus, intrusions and negative posttrauma cognitions and emotions might serve important roles in shaping and sustaining the PTSD symptom network. Symptom centrality was not entirely consistent across subgroups, supporting the heterogeneity of PTSD networks noted in previous reviews (Birkeland et al., 2020; Isvoranu et al., 2021). Future studies should test theory‐driven hypotheses to better understand the heterogeneity of PTSD etiology and treatment.

Consistent with previous longitudinal observational (Ge et al., 2019; Liang et al., 2021) and treatment studies (Schlesselmann et al., 2025; Xu et al., 2024), PTSD symptom structure remained stable while global symptom connectivity increased over time. The stable structure, as well as similar strongly connected pairs at both pre‐ and posttreatment, might reflect general psychological processes rather than PTSD‐specific pathological processes. For example, psychological and physiological reactions naturally co‐occur. Future studies should directly compare clinical and community populations to empirically test this hypothesis and potentially isolate PTSD‐specific pathological processes.

The increase in symptom connectivity with decreased symptom severity seems contrary to the network theory hypothesis, which posits that psychopathology arises from symptom interactions; however, this pattern potentially reflects a methodological artifact. When symptom scores on the 5‐point Likert scale move towards the lower end of the scale, reduced within‐person variation can inflate interitem connectivity. Improved research designs and statistical procedures to model network “activation” and “deactivation” processes are needed.

Key distinctions should be highlighted between undirected static centrality in cross‐sectional networks and directed dynamic centrality in temporal networks. Static central symptoms might not drive overall network change (Bringmann et al., 2019; Rodebaugh et al., 2018). For example, detachment consistently appeared central cross‐sectionally but was high in‐EI and low out‐EI longitudinally. In other words, detachment appeared to be sensitive to other symptoms cross‐sectionally and longitudinally but did not itself exert a strong influence on other symptoms. Internal and external avoidance exhibited low centrality cross‐sectionally but were high in‐EI and low out‐EI longitudinally, suggesting that avoidance may emerge in response to other symptoms rather than initiate changes across the network. The important role of physiological reactivity as a symptom that drives other symptoms over time is consistent with prior longitudinal network studies of exposure therapy (Hoffart et al., 2019). In the present study, physiological reactivity had a particularly strong impact on avoidance. The pathway from reckless behavior to negative beliefs, along with the persistence of negative beliefs, supported the previous finding that other PTSD symptoms more often drive changes in cognitions rather than the reverse (Benfer et al., 2021). However, the present CLPN results should be interpreted with caution due to low‐to‐moderate stability. Following Hoffart et al. (2019), collecting session‐by‐session data would provide a more nuanced understanding of the processes through which specific symptoms shift throughout treatment.

Symptom‐level analyses support previous findings (Graziano et al., 2024; Papini et al., 2020; Schlesselmann et al., 2025) showing stronger associations between changes in symptoms with higher pretreatment centrality and overall pre–post symptom change, above and beyond baseline severity. In addition, symptom severity itself was not correlated with centrality in the current study. These results underscore the unique predictive value of network‐based metrics. Improvements in central symptoms were related to improved posttreatment psychosocial functioning over and above changes in peripheral symptoms and demographic covariates. Note that SDQ data were missing for approximately 76% of participants, primarily due to administrative errors, including inconsistent administration across informants and missing assessments following treatment completion.

Central symptoms identified in our network analysis closely matched the key components of TF‐CBT. For example, exposure techniques are designed to reduce intrusion symptoms (Iyadurai et al., 2019; McNally, 2012). Similarly, cognitive coping techniques aim to directly alter negative cognitions, a central mechanism identified for TF‐CBT improvements (Alpert et al., 2023; Jensen et al., 2018; Pfeiffer et al., 2019). Additionally, conjoint parent–child sessions create a supportive environment, which might help youths reconnect with people and reduce feelings of detachment (Brown et al., 2020). With previous studies (Bringmann et al., 2019; Rodebaugh et al., 2018), we caution that centrality should not be equated to causality: Associations among symptoms are complex and may be bidirectional. Longitudinal randomized or strong quasi‐experimental designs (e.g., Hernán & Robins, 2020; Shadish et al., 2002) offer promise for strengthening causal inferences about the effects of treatment components on the underlying dynamics of the symptom network as well as clinical outcomes.

Finally, the present temporal network results provide preliminary new insight into symptom dynamics. In this study, out‐EI, the symptom's influence on other symptoms, was not strongly associated with either overall symptom‐level change or overall psychosocial functioning. In contrast, in‐EI was far more consistently associated with both symptom‐level change and psychosocial functioning. These patterns highlight that symptom influence and symptom susceptibility may serve distinct roles during treatment. For example, avoidance showed low out‐EI but high in‐EI, whereas physiological reactions showed high out‐EI but low in‐EI. Combined with the strong cross‐lagged effect from physiological reactions to internal avoidance, this pattern suggests that targeting physiological reactions may help reduce avoidance, and decreases in avoidance could serve as a proxy for treatment success. However, the present results should be interpreted with caution given that in‐EI, out‐EI, and clinical outcomes were based on two‐wave data. High in‐EI may simply reflect higher reactivity after overall symptom reductions, whereas high out‐EI may reflect early initiators of change. We encourage future researchers to incorporate multiple assessments (e.g., pretreatment, midtreatment, posttreatment, and follow‐up or, ideally, intensive longitudinal session‐by‐session data) to examine whether high out‐EI predicts subsequent symptom reductions or longer‐term functional improvement.

The present study has several limitations. First, as a single‐group pretest–posttest design, we cannot definitively rule out several threats to internal validity, such as maturation (Shadish et al., 2002). A randomized trial or observational study with a nontreated comparison group would greatly strengthen causal inferences. Second, two‐wave data have lower accuracy, power, and reliability than multiwave data or designs with intense longitudinal measurement (e.g., Parsons & McCormick, 2024). CLPN adds information beyond cross‐sectional networks by modeling directional associations across time, but multiwave data are needed to more accurately capture symptom dynamics. Third, group‐level network analysis may not capture heterogeneity in the PTSD network; idiographic (*n* = 1) network approaches with intensive longitudinal data (Bridges‐Curry, 2025; Reeves & Fisher, 2020) might be particularly useful for understanding heterogeneity. Fourth, experimental or strong quasi‐experimental designs are needed to test whether targeting central symptoms in the intervention actually drives improvement (Bringmann et al., 2019; Hernán & Robins, 2020; Rodebaugh et al., 2018; Shadish et al., 2002). Fifth, only trauma‐exposed youth who completed TF‐CBT and provided PTSD data at both time points were included in the analyses. Treatment dropout in our sample was related to trauma type and arousal severity (Swift, 2024), potentially limiting generalization. Sixth, intervention (e.g., dosage, fidelity) and therapist‐level information were not available; variability in those factors may have influenced the results. Seventh, the SDQ outcome measure demonstrated only minimally adequate internal consistency in this and other samples. Finally, many of the included youth experienced non–*DSM‐5* Criterion A events (e.g., bereavement, separation).

This exploratory study is the first to apply both cross‐sectional and temporal network analyses to examine PTSD symptom change upon TF‐CBT completion in youth using a large multiyear, state‐wide implementation. Cross‐sectional networks identified central symptoms at a given time point; participants with high pretreatment centrality may serve as important targets for treatment planning. Temporal networks captured directional influences between symptoms over time, offering insight into potential mechanisms of change. These approaches provide complementary perspectives, reinforcing the idea that PTSD is best understood as a network of interrelated symptoms rather than a single, homogeneous construct. Future research should expand on this work by collecting multiwave or intensive longitudinal data and employing experimental or high‐quality quasi‐experimental designs to strengthen causal inferences about how the symptom network changes during treatment. These advancements may ultimately help refine and personalize trauma interventions.

## AUTHOR NOTE

Sydni A. J. Basha is supported by the National Institutes of Health (T32 DA039772).

The authors would like to express sincere gratitude to all the clinicians and families who participated in the study. We also thank the Minnesota Department of Human Services (MN‐DHS) for providing funding for the statewide implementation of trauma‐focused cognitive behavioral therapy, though not  for this study, and to the individual agencies that participated. We would also like to thank Aditi Gupta for providing invaluable clinical insights.

## OPEN PRACTICES STATEMENT

The study reported in this article was not formally preregistered. The data have not been made available on a permanent third‐part archive; requests for the data may be sent via email to the corresponding author at abigail.gewirtz@asu.edu.

## AUTHOR CONTRIBUTIONS

Qiyue Cai: Conceptualization; Methodology; Formal analysis; Writing ‐ original draft; Writing ‐ review & editing. Bingyu Xu: Conceptualization; Methodology; Writing ‐ review & editing; Writing ‐ original draft. Sydni A. J. Basha: Writing ‐ original draft. SunKyung Lee: Data curation; Writing ‐ review & editing. Stephen G. West: Conceptualization; Methodology; Writing ‐ review & editing. Abigail H. Gewirtz: Writing ‐ review & editing.

## Supporting information



Supporting Information
