# Supplementary material for: Posttraumatic stress disorder symptom change in youth after trauma‐focused cognitive behavioral therapy: Insights from cross‐sectional and cross‐lagged panel network analysis
Source: J Trauma Stress. 2025 Dec 24;39(2):225–38. doi: 10.1002/jts.70030 (PMC13044382; doi:10.1002/jts.70030)
Supplement: Supplementary file 1 — Supporting Information [file JTS-39-225-s001.docx]

**Table S1.**

*Symptom severity scores pre- and post-treatment*

| Symptom Clusters | Pre-Treatment | | | Post-Treatment | | | Paired *t*-test | *P* value | Cohen's *d* |
| --- | --- | --- | --- | --- | --- | --- | --- | --- | --- |
|  | *n* | *M* (*SD*) | Range | *n* | *M* (*SD*) | Range |  |  |  |
| Intrusion (Cluster B) | 652 | 8.54 (5.41) | [0,20] | 652 | 3.79 (4.00) | [0,20] | 22.38 | < .001 | 0.88 |
| Avoidance (Cluster C) | 652 | 4.31 (2.47) | [0,8] | 652 | 2.11 (2.14) | [0,8] | 19.92 | < .001 | 0.78 |
| Negative alteration in  emotions and cognitions  (Cluster D) | 652 | 12.43 (6.57) | [0,28] | 652 | 7.06 (5.53) | [0,28] | 21.26 | < .001 | 0.83 |
| Hyperarousal (Cluster E) | 652 | 11.13 (5.12) | [0,24] | 652 | 6.93 (4.68) | [0,24] | 19.45 | < .001 | 0.76 |
| Total PTSD-RI-5 | 652 | 36.40 (16.33) | [3,77] | 652 | 19.89 (14.05) | [0,72] | 25.37 | < .001 | 0.99 |
| SDQ Total Difficulty | 152 | 18.71 (7.28) | [2,33] | 375 | 16.16 (6.78) | [1,35] | 3.55 | < .001 | 0.33 |

Note. Cohen’s (1988) *d* is a standardized effect size measure. 0.2, 0.5, and 0.8 represent small, medium, and large effect sizes, respectively.

PTSD-RI is the UCLA PTSD Reaction Index for the Diagnostic and Statistical Manual, Version 5 (Kaplow et al., 2020). Four clusters of PTSD symptoms (B, C, D, E) are identified. SDQ is the Strengths and Difficulties Questionnaire total difficulties (Goodman, 2001). The reduced n for this measure reflects missing data. A total of 500 participants were not administered or did not complete the SDQ.

**Table S2**

*Estimated edge values and 95% confidential interval (CI) width* *in cross-sectional and cross-lagged panel network models with 1,000 bootstrapping samples*

| N1 | N2 | Pre-Network | | Post-Network | | CLPN N1->N2  (unadjusted) | | CLPN N2->N1  (unadjusted) | | CLPN N1->N2  (adjusted) | | CLPN N2->N1  (adjusted) | |
| --- | --- | --- | --- | --- | --- | --- | --- | --- | --- | --- | --- | --- | --- |
|  |  | Mean (SD) | CI width | Mean (SD) | CI width | Mean (SD) | CI width | Mean (SD) | CI width | Mean (SD) | CI width | Mean (SD) | CI width |
| B1 | B2 | 0.16 (0.04) | 0.17 | 0.15 (0.06) | 0.23 | 0.05 (0.04) | 0.15 | 0 (0.02) | 0.08 | 0.05 (0.04) | 0.15 | 0 (0.02) | 0.09 |
| B1 | B3 | 0.21 (0.05) | 0.18 | 0.18 (0.06) | 0.23 | 0.02 (0.02) | 0.09 | 0.03 (0.03) | 0.13 | 0.02 (0.02) | 0.09 | 0.03 (0.03) | 0.13 |
| B1 | B4 | 0.14 (0.05) | 0.18 | 0.07 (0.05) | 0.21 | 0.05 (0.04) | 0.16 | -0.01 (0.03) | 0.11 | 0.04 (0.04) | 0.15 | -0.02 (0.03) | 0.12 |
| B1 | B5 | 0.2 (0.04) | 0.18 | 0.16 (0.05) | 0.22 | -0.01 (0.03) | 0.11 | 0.06 (0.04) | 0.14 | -0.01 (0.03) | 0.12 | 0.07 (0.04) | 0.16 |
| B1 | C1 | 0.03 (0.03) | 0.14 | 0.07 (0.05) | 0.19 | 0.02 (0.03) | 0.13 | 0 (0.01) | 0.05 | 0.02 (0.03) | 0.13 | 0 (0.01) | 0.05 |
| B1 | C2 | 0.03 (0.03) | 0.12 | 0.04 (0.04) | 0.17 | 0.05 (0.04) | 0.16 | 0.01 (0.02) | 0.07 | 0.04 (0.04) | 0.15 | 0.01 (0.02) | 0.07 |
| B1 | D1 | 0 (0.01) | 0.04 | 0.08 (0.05) | 0.19 | 0.02 (0.03) | 0.13 | 0.02 (0.02) | 0.09 | 0.02 (0.03) | 0.12 | 0.02 (0.02) | 0.09 |
| B1 | D2 | 0 (0.01) | 0.04 | 0.03 (0.04) | 0.15 | 0.05 (0.04) | 0.16 | -0.01 (0.02) | 0.08 | 0.04 (0.04) | 0.16 | -0.01 (0.02) | 0.08 |
| B1 | D3 | 0.06 (0.04) | 0.16 | 0.07 (0.05) | 0.18 | 0 (0.03) | 0.11 | 0 (0.02) | 0.06 | -0.01 (0.03) | 0.13 | 0 (0.01) | 0.06 |
| B1 | D4 | 0.04 (0.04) | 0.14 | 0.05 (0.04) | 0.17 | 0 (0.03) | 0.11 | 0.02 (0.02) | 0.1 | 0 (0.03) | 0.11 | 0.03 (0.03) | 0.1 |
| B1 | D5 | 0 (0.01) | 0.03 | 0.01 (0.02) | 0.1 | 0.01 (0.03) | 0.11 | 0.05 (0.03) | 0.14 | 0.01 (0.02) | 0.09 | 0.06 (0.04) | 0.14 |
| B1 | D6 | 0.13 (0.04) | 0.17 | 0.06 (0.05) | 0.18 | 0.01 (0.03) | 0.11 | 0 (0.02) | 0.07 | 0.01 (0.02) | 0.1 | 0 (0.02) | 0.08 |
| B1 | D7 | 0.01 (0.02) | 0.08 | 0.01 (0.02) | 0.08 | 0.01 (0.02) | 0.09 | -0.01 (0.02) | 0.09 | 0.01 (0.02) | 0.09 | -0.01 (0.02) | 0.09 |
| B1 | E1 | -0.03 (0.04) | 0.14 | 0 (0.01) | 0.05 | 0.01 (0.03) | 0.11 | 0 (0.02) | 0.08 | 0.01 (0.03) | 0.11 | -0.01 (0.02) | 0.09 |
| B1 | E2 | 0 (0.01) | 0.05 | 0 (0.02) | 0.1 | -0.01 (0.02) | 0.07 | 0.02 (0.02) | 0.1 | -0.01 (0.02) | 0.07 | 0.02 (0.03) | 0.11 |
| B1 | E3 | 0.01 (0.02) | 0.08 | 0.03 (0.04) | 0.15 | 0.02 (0.03) | 0.11 | 0 (0.02) | 0.07 | 0.02 (0.03) | 0.12 | 0 (0.02) | 0.07 |
| B1 | E4 | 0.05 (0.04) | 0.15 | 0.03 (0.04) | 0.14 | 0 (0.02) | 0.09 | 0 (0.02) | 0.07 | 0 (0.03) | 0.11 | -0.01 (0.02) | 0.08 |
| B1 | E5 | 0.01 (0.02) | 0.07 | 0.02 (0.03) | 0.11 | -0.04 (0.04) | 0.16 | 0 (0.02) | 0.07 | -0.04 (0.04) | 0.16 | 0.01 (0.02) | 0.08 |
| B1 | E6 | 0.01 (0.02) | 0.07 | 0.1 (0.05) | 0.19 | -0.02 (0.04) | 0.16 | 0.06 (0.03) | 0.12 | -0.02 (0.04) | 0.14 | 0.06 (0.03) | 0.12 |
| B2 | B3 | 0.05 (0.04) | 0.16 | 0.11 (0.06) | 0.23 | -0.01 (0.02) | 0.08 | 0 (0.02) | 0.08 | -0.01 (0.02) | 0.09 | 0 (0.02) | 0.08 |
| B2 | B4 | 0.15 (0.04) | 0.17 | 0.13 (0.05) | 0.18 | 0.02 (0.03) | 0.11 | -0.01 (0.02) | 0.1 | 0.02 (0.03) | 0.12 | -0.01 (0.03) | 0.1 |
| B2 | B5 | 0.04 (0.04) | 0.14 | 0.01 (0.02) | 0.09 | -0.01 (0.02) | 0.1 | 0.03 (0.03) | 0.12 | -0.01 (0.02) | 0.1 | 0.03 (0.03) | 0.12 |
| B2 | C1 | 0 (0.01) | 0.04 | 0.08 (0.04) | 0.18 | -0.03 (0.04) | 0.15 | -0.01 (0.02) | 0.08 | -0.02 (0.04) | 0.14 | -0.01 (0.02) | 0.08 |
| B2 | C2 | 0.03 (0.03) | 0.13 | 0 (0.02) | 0.07 | -0.01 (0.03) | 0.11 | 0 (0.02) | 0.06 | -0.01 (0.02) | 0.09 | 0 (0.02) | 0.06 |
| B2 | D1 | 0.01 (0.02) | 0.07 | 0.01 (0.02) | 0.1 | -0.05 (0.03) | 0.14 | 0.04 (0.03) | 0.11 | -0.05 (0.03) | 0.14 | 0.04 (0.03) | 0.12 |
| B2 | D2 | 0.02 (0.02) | 0.1 | 0.03 (0.03) | 0.13 | -0.05 (0.04) | 0.16 | 0.01 (0.02) | 0.09 | -0.05 (0.04) | 0.17 | 0.01 (0.02) | 0.1 |
| B2 | D3 | 0.02 (0.02) | 0.1 | 0 (0.01) | 0.05 | -0.02 (0.03) | 0.13 | 0.02 (0.02) | 0.09 | -0.01 (0.03) | 0.12 | 0.02 (0.02) | 0.09 |
| B2 | D4 | 0.05 (0.04) | 0.15 | 0.03 (0.04) | 0.14 | -0.03 (0.04) | 0.14 | 0.01 (0.02) | 0.09 | -0.03 (0.03) | 0.14 | 0.01 (0.02) | 0.09 |
| B2 | D5 | 0 (0.01) | 0.04 | 0 (0.01) | 0.05 | -0.05 (0.03) | 0.12 | 0.07 (0.04) | 0.15 | -0.04 (0.03) | 0.11 | 0.07 (0.04) | 0.16 |
| B2 | D6 | 0 (0.01) | 0.05 | 0 (0.01) | 0.05 | -0.06 (0.04) | 0.15 | 0 (0.02) | 0.08 | -0.05 (0.04) | 0.14 | 0 (0.02) | 0.08 |
| B2 | D7 | 0.02 (0.02) | 0.1 | 0.03 (0.03) | 0.13 | -0.05 (0.03) | 0.14 | 0 (0.02) | 0.09 | -0.05 (0.03) | 0.13 | 0 (0.02) | 0.09 |
| B2 | E1 | 0.01 (0.02) | 0.07 | 0 (0.01) | 0.06 | 0 (0.02) | 0.08 | -0.01 (0.02) | 0.09 | -0.01 (0.02) | 0.1 | -0.01 (0.02) | 0.09 |
| B2 | E2 | 0.04 (0.04) | 0.15 | 0.02 (0.03) | 0.14 | -0.01 (0.02) | 0.08 | 0.02 (0.03) | 0.11 | -0.01 (0.02) | 0.08 | 0.02 (0.03) | 0.1 |
| B2 | E3 | 0.08 (0.04) | 0.17 | 0.11 (0.05) | 0.19 | 0 (0.02) | 0.07 | 0.01 (0.02) | 0.08 | 0 (0.02) | 0.07 | 0.01 (0.02) | 0.08 |
| B2 | E4 | 0 (0.01) | 0.06 | -0.01 (0.03) | 0.11 | -0.01 (0.03) | 0.1 | -0.01 (0.02) | 0.08 | -0.01 (0.03) | 0.1 | -0.01 (0.02) | 0.08 |
| B2 | E5 | 0.01 (0.02) | 0.08 | 0.01 (0.01) | 0.06 | -0.03 (0.03) | 0.13 | 0.01 (0.02) | 0.08 | -0.02 (0.03) | 0.12 | 0.01 (0.02) | 0.09 |
| B2 | E6 | 0.19 (0.04) | 0.18 | 0.15 (0.05) | 0.19 | -0.01 (0.03) | 0.12 | 0.05 (0.03) | 0.12 | -0.01 (0.03) | 0.12 | 0.05 (0.03) | 0.12 |
| B3 | B4 | 0.07 (0.05) | 0.18 | 0.15 (0.05) | 0.2 | 0 (0.02) | 0.09 | 0 (0.02) | 0.07 | 0.01 (0.02) | 0.09 | 0 (0.02) | 0.07 |
| B3 | B5 | 0.07 (0.04) | 0.18 | 0.12 (0.06) | 0.22 | 0.07 (0.04) | 0.17 | 0.01 (0.02) | 0.09 | 0.07 (0.04) | 0.18 | 0.02 (0.02) | 0.09 |
| B3 | C1 | 0.01 (0.02) | 0.07 | 0.05 (0.05) | 0.19 | 0 (0.03) | 0.11 | -0.02 (0.02) | 0.09 | 0 (0.02) | 0.1 | -0.02 (0.02) | 0.09 |
| B3 | C2 | 0.09 (0.05) | 0.18 | 0.11 (0.05) | 0.22 | 0.05 (0.04) | 0.16 | 0 (0.01) | 0.06 | 0.05 (0.04) | 0.16 | 0 (0.01) | 0.06 |
| B3 | D1 | 0 (0.01) | 0.05 | 0.03 (0.04) | 0.16 | -0.03 (0.03) | 0.14 | 0.03 (0.02) | 0.09 | -0.03 (0.04) | 0.14 | 0.03 (0.02) | 0.1 |
| B3 | D2 | 0 (0.01) | 0.04 | 0.01 (0.03) | 0.1 | 0.01 (0.03) | 0.12 | 0.01 (0.02) | 0.08 | 0.01 (0.03) | 0.11 | 0.01 (0.02) | 0.08 |
| B3 | D3 | 0.07 (0.04) | 0.17 | 0.05 (0.05) | 0.18 | 0 (0.03) | 0.13 | 0 (0.01) | 0.05 | 0 (0.03) | 0.12 | 0 (0.01) | 0.05 |
| B3 | D4 | 0.14 (0.05) | 0.19 | 0.01 (0.02) | 0.1 | 0 (0.03) | 0.12 | 0.02 (0.02) | 0.09 | 0 (0.03) | 0.12 | 0.02 (0.02) | 0.09 |
| B3 | D5 | 0.01 (0.01) | 0.06 | 0.04 (0.04) | 0.15 | -0.02 (0.03) | 0.11 | 0.04 (0.03) | 0.12 | -0.02 (0.03) | 0.1 | 0.04 (0.03) | 0.12 |
| B3 | D6 | 0.02 (0.03) | 0.11 | 0.01 (0.02) | 0.07 | 0.03 (0.03) | 0.12 | -0.01 (0.02) | 0.09 | 0.03 (0.03) | 0.12 | -0.01 (0.02) | 0.09 |
| B3 | D7 | 0.01 (0.02) | 0.09 | 0.01 (0.02) | 0.07 | 0.02 (0.03) | 0.1 | 0.01 (0.02) | 0.08 | 0.02 (0.02) | 0.1 | 0.01 (0.02) | 0.07 |
| B3 | E1 | 0 (0.02) | 0.06 | 0.03 (0.04) | 0.15 | 0 (0.02) | 0.08 | -0.01 (0.02) | 0.06 | 0 (0.02) | 0.08 | 0 (0.02) | 0.06 |
| B3 | E2 | -0.01 (0.02) | 0.08 | 0.06 (0.05) | 0.2 | 0.01 (0.02) | 0.09 | 0.01 (0.02) | 0.07 | 0.01 (0.02) | 0.09 | 0.01 (0.02) | 0.07 |
| B3 | E3 | 0.1 (0.05) | 0.18 | 0.11 (0.05) | 0.22 | 0 (0.02) | 0.08 | 0.02 (0.02) | 0.09 | 0 (0.02) | 0.08 | 0.02 (0.02) | 0.08 |
| B3 | E4 | 0.03 (0.03) | 0.14 | 0.01 (0.02) | 0.08 | 0.02 (0.03) | 0.13 | 0.03 (0.02) | 0.09 | 0.04 (0.04) | 0.16 | 0.03 (0.02) | 0.09 |
| B3 | E5 | 0.01 (0.02) | 0.08 | 0 (0.02) | 0.06 | 0.04 (0.04) | 0.17 | 0.02 (0.02) | 0.08 | 0.03 (0.04) | 0.16 | 0.02 (0.02) | 0.09 |
| B3 | E6 | 0.01 (0.02) | 0.1 | 0 (0.02) | 0.07 | 0.03 (0.04) | 0.16 | 0.01 (0.02) | 0.07 | 0.03 (0.04) | 0.16 | 0.01 (0.02) | 0.07 |
| B4 | B5 | 0.25 (0.05) | 0.18 | 0.22 (0.05) | 0.21 | 0.03 (0.04) | 0.14 | 0.06 (0.04) | 0.15 | 0.03 (0.04) | 0.14 | 0.06 (0.04) | 0.15 |
| B4 | C1 | 0.13 (0.04) | 0.18 | 0.08 (0.05) | 0.2 | 0.01 (0.03) | 0.1 | -0.02 (0.03) | 0.1 | 0.01 (0.03) | 0.1 | -0.02 (0.03) | 0.1 |
| B4 | C2 | 0.14 (0.04) | 0.18 | 0.17 (0.05) | 0.19 | 0.01 (0.03) | 0.13 | 0 (0.02) | 0.07 | 0.01 (0.03) | 0.13 | 0 (0.02) | 0.08 |
| B4 | D1 | 0.02 (0.03) | 0.1 | 0.06 (0.05) | 0.19 | -0.02 (0.03) | 0.13 | 0.05 (0.03) | 0.13 | -0.02 (0.03) | 0.13 | 0.05 (0.03) | 0.14 |
| B4 | D2 | 0.01 (0.02) | 0.07 | 0.06 (0.04) | 0.16 | 0.01 (0.03) | 0.1 | 0.01 (0.03) | 0.1 | 0.01 (0.03) | 0.11 | 0.01 (0.02) | 0.1 |
| B4 | D3 | 0.04 (0.04) | 0.15 | 0.09 (0.05) | 0.19 | 0.01 (0.03) | 0.13 | 0.01 (0.02) | 0.08 | 0.01 (0.03) | 0.12 | 0.01 (0.02) | 0.08 |
| B4 | D4 | 0.01 (0.02) | 0.09 | 0 (0.01) | 0.05 | -0.04 (0.05) | 0.18 | 0.05 (0.04) | 0.14 | -0.04 (0.05) | 0.2 | 0.04 (0.03) | 0.14 |
| B4 | D5 | 0 (0.01) | 0.03 | -0.02 (0.03) | 0.13 | -0.05 (0.04) | 0.15 | 0.02 (0.03) | 0.12 | -0.05 (0.04) | 0.14 | 0.02 (0.03) | 0.12 |
| B4 | D6 | 0.03 (0.03) | 0.13 | 0 (0.01) | 0.04 | 0 (0.03) | 0.11 | -0.01 (0.03) | 0.12 | -0.01 (0.03) | 0.11 | -0.01 (0.03) | 0.12 |
| B4 | D7 | 0.01 (0.02) | 0.08 | 0.02 (0.03) | 0.11 | -0.04 (0.04) | 0.15 | 0.04 (0.04) | 0.14 | -0.04 (0.04) | 0.15 | 0.03 (0.03) | 0.13 |
| B4 | E1 | 0.03 (0.03) | 0.12 | 0.01 (0.02) | 0.09 | -0.01 (0.03) | 0.1 | 0 (0.02) | 0.08 | -0.01 (0.03) | 0.11 | 0.01 (0.02) | 0.08 |
| B4 | E2 | -0.03 (0.03) | 0.13 | -0.03 (0.04) | 0.16 | -0.05 (0.03) | 0.13 | 0 (0.02) | 0.09 | -0.03 (0.03) | 0.13 | 0 (0.02) | 0.09 |
| B4 | E3 | 0.01 (0.02) | 0.06 | 0 (0.01) | 0.05 | -0.04 (0.04) | 0.17 | 0 (0.02) | 0.08 | -0.03 (0.04) | 0.17 | 0 (0.02) | 0.07 |
| B4 | E4 | 0.04 (0.04) | 0.15 | 0.03 (0.03) | 0.13 | -0.05 (0.05) | 0.18 | 0 (0.02) | 0.08 | -0.07 (0.05) | 0.19 | 0 (0.02) | 0.07 |
| B4 | E5 | 0.04 (0.04) | 0.14 | -0.02 (0.03) | 0.13 | -0.04 (0.04) | 0.16 | -0.03 (0.03) | 0.13 | -0.04 (0.04) | 0.16 | -0.03 (0.03) | 0.13 |
| B4 | E6 | 0.03 (0.03) | 0.13 | 0 (0.01) | 0.05 | -0.07 (0.05) | 0.22 | 0.01 (0.02) | 0.08 | -0.08 (0.06) | 0.23 | 0.01 (0.02) | 0.09 |
| B5 | C1 | 0.16 (0.04) | 0.18 | 0.12 (0.05) | 0.21 | 0.1 (0.05) | 0.18 | -0.08 (0.04) | 0.14 | 0.1 (0.05) | 0.18 | -0.08 (0.03) | 0.14 |
| B5 | C2 | 0.13 (0.04) | 0.18 | 0.06 (0.05) | 0.18 | 0.07 (0.04) | 0.15 | 0.01 (0.02) | 0.1 | 0.07 (0.04) | 0.16 | 0.01 (0.02) | 0.09 |
| B5 | D1 | 0.01 (0.02) | 0.08 | 0.04 (0.04) | 0.16 | 0.04 (0.03) | 0.14 | 0.04 (0.03) | 0.13 | 0.03 (0.03) | 0.13 | 0.04 (0.03) | 0.13 |
| B5 | D2 | 0.01 (0.02) | 0.06 | 0 (0.01) | 0.04 | -0.01 (0.02) | 0.09 | 0 (0.03) | 0.11 | -0.01 (0.03) | 0.1 | 0 (0.03) | 0.1 |
| B5 | D3 | 0.04 (0.04) | 0.16 | 0.12 (0.05) | 0.21 | 0.02 (0.03) | 0.13 | 0 (0.02) | 0.08 | 0.03 (0.03) | 0.14 | 0 (0.02) | 0.08 |
| B5 | D4 | 0.04 (0.04) | 0.14 | 0.07 (0.04) | 0.18 | 0.06 (0.05) | 0.18 | 0.1 (0.04) | 0.14 | 0.07 (0.05) | 0.19 | 0.1 (0.04) | 0.15 |
| B5 | D5 | 0.04 (0.03) | 0.13 | 0.03 (0.03) | 0.13 | 0.04 (0.03) | 0.13 | 0.04 (0.04) | 0.15 | 0.04 (0.03) | 0.13 | 0.04 (0.04) | 0.14 |
| B5 | D6 | 0 (0.01) | 0.03 | 0.05 (0.04) | 0.16 | 0.03 (0.03) | 0.11 | 0.01 (0.03) | 0.1 | 0.03 (0.03) | 0.12 | 0.01 (0.03) | 0.1 |
| B5 | D7 | 0.05 (0.03) | 0.13 | 0 (0.02) | 0.06 | 0.02 (0.03) | 0.11 | 0.01 (0.03) | 0.12 | 0.02 (0.03) | 0.1 | 0 (0.03) | 0.11 |
| B5 | E1 | 0 (0.01) | 0.05 | -0.01 (0.02) | 0.09 | 0 (0.02) | 0.07 | -0.05 (0.03) | 0.13 | 0 (0.02) | 0.08 | -0.04 (0.03) | 0.13 |
| B5 | E2 | 0 (0.01) | 0.05 | 0.07 (0.05) | 0.21 | 0.01 (0.02) | 0.07 | 0.05 (0.04) | 0.15 | 0.01 (0.02) | 0.08 | 0.05 (0.04) | 0.16 |
| B5 | E3 | 0.01 (0.02) | 0.07 | -0.01 (0.02) | 0.08 | 0 (0.02) | 0.08 | -0.02 (0.03) | 0.11 | 0 (0.02) | 0.08 | -0.02 (0.03) | 0.11 |
| B5 | E4 | 0.02 (0.03) | 0.1 | 0.03 (0.03) | 0.13 | 0 (0.02) | 0.08 | 0 (0.02) | 0.09 | -0.01 (0.02) | 0.09 | 0 (0.02) | 0.08 |
| B5 | E5 | 0.01 (0.02) | 0.08 | 0 (0.01) | 0.06 | 0 (0.03) | 0.1 | -0.02 (0.03) | 0.1 | 0 (0.02) | 0.09 | -0.02 (0.03) | 0.11 |
| B5 | E6 | 0.03 (0.03) | 0.13 | 0 (0.01) | 0.05 | 0.1 (0.05) | 0.21 | 0.04 (0.03) | 0.12 | 0.08 (0.05) | 0.21 | 0.04 (0.03) | 0.12 |
| C1 | C2 | 0.07 (0.04) | 0.17 | 0.19 (0.05) | 0.22 | 0 (0.02) | 0.08 | 0 (0.02) | 0.09 | 0 (0.02) | 0.07 | 0 (0.02) | 0.09 |
| C1 | D1 | 0.01 (0.02) | 0.09 | 0.01 (0.02) | 0.09 | 0.01 (0.02) | 0.08 | 0.03 (0.03) | 0.13 | 0.01 (0.02) | 0.08 | 0.03 (0.03) | 0.13 |
| C1 | D2 | 0.01 (0.02) | 0.07 | 0.02 (0.03) | 0.12 | -0.05 (0.04) | 0.15 | 0 (0.02) | 0.09 | -0.06 (0.04) | 0.14 | 0 (0.02) | 0.09 |
| C1 | D3 | 0.07 (0.04) | 0.18 | 0.05 (0.04) | 0.16 | -0.08 (0.04) | 0.16 | 0.04 (0.03) | 0.14 | -0.08 (0.04) | 0.15 | 0.04 (0.03) | 0.13 |
| C1 | D4 | 0.06 (0.04) | 0.18 | 0.09 (0.04) | 0.18 | -0.04 (0.04) | 0.15 | 0.02 (0.03) | 0.12 | -0.04 (0.03) | 0.14 | 0.01 (0.03) | 0.11 |
| C1 | D5 | 0.01 (0.02) | 0.08 | 0.02 (0.03) | 0.1 | -0.02 (0.02) | 0.09 | 0.03 (0.03) | 0.13 | -0.02 (0.02) | 0.09 | 0.03 (0.03) | 0.14 |
| C1 | D6 | 0.08 (0.04) | 0.17 | 0.02 (0.03) | 0.11 | -0.05 (0.03) | 0.13 | 0.07 (0.04) | 0.18 | -0.05 (0.03) | 0.12 | 0.08 (0.05) | 0.19 |
| C1 | D7 | 0 (0.01) | 0.04 | 0.1 (0.04) | 0.17 | -0.01 (0.02) | 0.08 | -0.01 (0.03) | 0.13 | -0.01 (0.02) | 0.07 | -0.02 (0.03) | 0.13 |
| C1 | E1 | 0.02 (0.02) | 0.1 | 0 (0.01) | 0.05 | -0.02 (0.03) | 0.1 | -0.04 (0.04) | 0.15 | -0.02 (0.02) | 0.1 | -0.04 (0.04) | 0.15 |
| C1 | E2 | 0.01 (0.02) | 0.07 | 0 (0.02) | 0.06 | -0.01 (0.02) | 0.07 | 0.03 (0.04) | 0.15 | -0.01 (0.01) | 0.06 | 0.03 (0.04) | 0.15 |
| C1 | E3 | 0.01 (0.02) | 0.07 | 0.01 (0.02) | 0.09 | 0 (0.02) | 0.07 | 0 (0.02) | 0.09 | 0 (0.02) | 0.06 | -0.01 (0.02) | 0.09 |
| C1 | E4 | 0.02 (0.02) | 0.1 | 0.02 (0.03) | 0.11 | -0.01 (0.03) | 0.1 | 0.01 (0.02) | 0.1 | -0.02 (0.03) | 0.11 | 0.01 (0.02) | 0.1 |
| C1 | E5 | 0 (0.01) | 0.04 | 0.01 (0.02) | 0.08 | -0.05 (0.04) | 0.14 | 0.02 (0.03) | 0.11 | -0.04 (0.03) | 0.14 | 0.02 (0.03) | 0.12 |
| C1 | E6 | 0 (0.01) | 0.05 | 0.01 (0.02) | 0.1 | -0.03 (0.04) | 0.14 | 0.01 (0.02) | 0.09 | -0.02 (0.03) | 0.13 | 0.02 (0.03) | 0.1 |
| C2 | D1 | 0.01 (0.02) | 0.08 | 0.02 (0.03) | 0.11 | 0.02 (0.02) | 0.1 | 0.07 (0.04) | 0.14 | 0.01 (0.02) | 0.09 | 0.07 (0.04) | 0.15 |
| C2 | D2 | 0.04 (0.04) | 0.14 | 0 (0.02) | 0.06 | 0.01 (0.02) | 0.09 | -0.01 (0.02) | 0.1 | 0.01 (0.02) | 0.1 | -0.01 (0.03) | 0.1 |
| C2 | D3 | 0.01 (0.02) | 0.1 | 0.05 (0.04) | 0.17 | 0.04 (0.04) | 0.14 | 0.03 (0.03) | 0.12 | 0.03 (0.03) | 0.13 | 0.03 (0.03) | 0.11 |
| C2 | D4 | 0.08 (0.05) | 0.19 | 0.06 (0.04) | 0.17 | 0.04 (0.03) | 0.14 | 0.04 (0.04) | 0.15 | 0.03 (0.03) | 0.13 | 0.04 (0.04) | 0.14 |
| C2 | D5 | 0.01 (0.02) | 0.09 | 0 (0.01) | 0.05 | 0.01 (0.02) | 0.08 | 0.01 (0.02) | 0.1 | 0 (0.02) | 0.06 | 0.01 (0.03) | 0.1 |
| C2 | D6 | 0 (0.01) | 0.05 | -0.01 (0.02) | 0.08 | 0.02 (0.03) | 0.1 | -0.01 (0.03) | 0.1 | 0.02 (0.02) | 0.09 | -0.01 (0.02) | 0.09 |
| C2 | D7 | 0.01 (0.02) | 0.06 | -0.02 (0.03) | 0.11 | 0.04 (0.03) | 0.1 | 0 (0.02) | 0.09 | 0.03 (0.03) | 0.1 | 0 (0.02) | 0.08 |
| C2 | E1 | 0 (0.01) | 0.06 | 0.01 (0.02) | 0.09 | -0.02 (0.02) | 0.09 | 0.01 (0.03) | 0.1 | -0.02 (0.02) | 0.09 | 0.01 (0.03) | 0.1 |
| C2 | E2 | 0 (0.01) | 0.05 | -0.02 (0.03) | 0.13 | 0 (0.01) | 0.05 | -0.04 (0.03) | 0.14 | 0 (0.01) | 0.05 | -0.03 (0.03) | 0.14 |
| C2 | E3 | 0.14 (0.05) | 0.18 | 0.08 (0.05) | 0.2 | 0.03 (0.03) | 0.12 | 0.02 (0.03) | 0.11 | 0.03 (0.03) | 0.12 | 0.01 (0.02) | 0.09 |
| C2 | E4 | 0.03 (0.03) | 0.14 | 0 (0.01) | 0.06 | 0 (0.02) | 0.08 | 0 (0.02) | 0.08 | 0 (0.02) | 0.09 | 0.01 (0.02) | 0.08 |
| C2 | E5 | -0.02 (0.03) | 0.11 | 0.01 (0.02) | 0.08 | -0.03 (0.03) | 0.12 | 0 (0.02) | 0.08 | -0.02 (0.03) | 0.12 | 0 (0.02) | 0.08 |
| C2 | E6 | 0 (0.02) | 0.07 | 0 (0.01) | 0.06 | -0.03 (0.03) | 0.13 | 0.01 (0.02) | 0.08 | -0.03 (0.03) | 0.13 | 0.01 (0.02) | 0.08 |
| D1 | D2 | 0 (0.01) | 0.04 | 0 (0.01) | 0.06 | 0 (0.02) | 0.09 | 0.08 (0.04) | 0.17 | 0 (0.02) | 0.09 | 0.07 (0.04) | 0.17 |
| D1 | D3 | 0.04 (0.04) | 0.14 | 0.04 (0.04) | 0.16 | 0.03 (0.03) | 0.12 | -0.01 (0.02) | 0.09 | 0.03 (0.03) | 0.12 | 0 (0.02) | 0.07 |
| D1 | D4 | 0.06 (0.04) | 0.16 | 0.03 (0.04) | 0.15 | 0 (0.03) | 0.1 | 0.01 (0.02) | 0.09 | 0 (0.02) | 0.09 | 0.01 (0.02) | 0.08 |
| D1 | D5 | 0.01 (0.02) | 0.06 | 0.06 (0.05) | 0.19 | 0.02 (0.02) | 0.09 | 0.05 (0.04) | 0.14 | 0.02 (0.02) | 0.09 | 0.05 (0.03) | 0.14 |
| D1 | D6 | 0.1 (0.04) | 0.18 | 0.01 (0.02) | 0.1 | 0.01 (0.02) | 0.09 | -0.03 (0.04) | 0.14 | 0 (0.02) | 0.08 | -0.03 (0.04) | 0.14 |
| D1 | D7 | 0.03 (0.03) | 0.14 | 0.07 (0.05) | 0.18 | 0.02 (0.02) | 0.09 | -0.01 (0.03) | 0.11 | 0.01 (0.02) | 0.08 | -0.01 (0.02) | 0.1 |
| D1 | E1 | -0.01 (0.02) | 0.08 | 0 (0.01) | 0.06 | 0 (0.02) | 0.06 | -0.01 (0.02) | 0.09 | 0.01 (0.02) | 0.07 | -0.01 (0.02) | 0.09 |
| D1 | E2 | 0.02 (0.03) | 0.12 | 0 (0.02) | 0.1 | 0 (0.02) | 0.06 | -0.03 (0.03) | 0.11 | 0 (0.01) | 0.06 | -0.02 (0.03) | 0.11 |
| D1 | E3 | -0.01 (0.02) | 0.07 | 0.07 (0.05) | 0.19 | 0.01 (0.02) | 0.09 | 0.08 (0.04) | 0.14 | 0.01 (0.02) | 0.08 | 0.07 (0.03) | 0.14 |
| D1 | E4 | 0.08 (0.04) | 0.18 | 0.04 (0.04) | 0.15 | 0.02 (0.03) | 0.1 | -0.02 (0.03) | 0.11 | 0.02 (0.03) | 0.11 | -0.02 (0.03) | 0.1 |
| D1 | E5 | 0 (0.01) | 0.06 | 0.02 (0.03) | 0.11 | -0.01 (0.02) | 0.1 | 0 (0.02) | 0.08 | -0.01 (0.02) | 0.1 | 0 (0.02) | 0.09 |
| D1 | E6 | 0.01 (0.02) | 0.09 | -0.02 (0.03) | 0.12 | -0.02 (0.03) | 0.12 | 0.01 (0.02) | 0.08 | -0.02 (0.03) | 0.12 | 0 (0.02) | 0.08 |
| D2 | D3 | 0.07 (0.04) | 0.17 | 0.08 (0.04) | 0.18 | 0.02 (0.03) | 0.12 | 0 (0.02) | 0.08 | 0.02 (0.03) | 0.13 | 0 (0.02) | 0.09 |
| D2 | D4 | 0.12 (0.04) | 0.18 | 0.14 (0.05) | 0.18 | 0.06 (0.04) | 0.18 | 0.03 (0.03) | 0.13 | 0.06 (0.05) | 0.19 | 0.03 (0.04) | 0.14 |
| D2 | D5 | 0.04 (0.04) | 0.16 | 0.06 (0.04) | 0.18 | -0.01 (0.02) | 0.09 | 0.01 (0.03) | 0.12 | -0.01 (0.02) | 0.09 | 0.02 (0.03) | 0.12 |
| D2 | D6 | 0.24 (0.04) | 0.18 | 0.2 (0.05) | 0.18 | 0.02 (0.03) | 0.11 | 0 (0.03) | 0.1 | 0.02 (0.03) | 0.11 | 0 (0.03) | 0.11 |
| D2 | D7 | 0.19 (0.04) | 0.18 | 0.12 (0.05) | 0.19 | 0.02 (0.02) | 0.09 | 0.04 (0.04) | 0.15 | 0.01 (0.02) | 0.08 | 0.03 (0.04) | 0.14 |
| D2 | E1 | 0.08 (0.04) | 0.16 | 0.05 (0.04) | 0.16 | -0.01 (0.02) | 0.09 | -0.02 (0.03) | 0.12 | -0.01 (0.03) | 0.11 | -0.02 (0.03) | 0.12 |
| D2 | E2 | 0.06 (0.04) | 0.17 | 0.05 (0.04) | 0.18 | 0.04 (0.03) | 0.12 | 0.1 (0.04) | 0.17 | 0.04 (0.03) | 0.12 | 0.1 (0.04) | 0.17 |
| D2 | E3 | 0.04 (0.04) | 0.15 | 0.07 (0.04) | 0.16 | 0.02 (0.03) | 0.11 | 0.02 (0.03) | 0.11 | 0.02 (0.03) | 0.11 | 0.02 (0.03) | 0.11 |
| D2 | E4 | 0.01 (0.02) | 0.09 | 0.02 (0.02) | 0.09 | -0.01 (0.02) | 0.09 | 0.02 (0.03) | 0.11 | 0 (0.02) | 0.1 | 0.03 (0.03) | 0.11 |
| D2 | E5 | 0.1 (0.04) | 0.16 | 0.08 (0.05) | 0.19 | 0.01 (0.03) | 0.13 | 0.04 (0.03) | 0.13 | 0 (0.03) | 0.12 | 0.04 (0.03) | 0.14 |
| D2 | E6 | 0.03 (0.03) | 0.13 | 0.02 (0.02) | 0.1 | -0.01 (0.03) | 0.14 | 0.02 (0.03) | 0.1 | 0 (0.03) | 0.13 | 0.02 (0.03) | 0.1 |
| D3 | D4 | 0.2 (0.05) | 0.19 | 0.21 (0.05) | 0.2 | 0.05 (0.04) | 0.15 | 0.1 (0.04) | 0.16 | 0.05 (0.04) | 0.16 | 0.09 (0.04) | 0.16 |
| D3 | D5 | 0.01 (0.02) | 0.07 | 0.03 (0.03) | 0.13 | -0.01 (0.02) | 0.08 | 0.04 (0.04) | 0.15 | -0.02 (0.02) | 0.08 | 0.04 (0.04) | 0.15 |
| D3 | D6 | 0.1 (0.04) | 0.18 | 0 (0.01) | 0.06 | 0 (0.02) | 0.08 | -0.01 (0.03) | 0.12 | 0 (0.02) | 0.08 | -0.02 (0.03) | 0.13 |
| D3 | D7 | 0.06 (0.04) | 0.16 | 0 (0.01) | 0.05 | 0.01 (0.02) | 0.06 | 0.02 (0.03) | 0.12 | 0.01 (0.02) | 0.07 | 0.02 (0.03) | 0.12 |
| D3 | E1 | 0.01 (0.02) | 0.08 | 0.01 (0.02) | 0.09 | 0 (0.02) | 0.07 | -0.03 (0.03) | 0.14 | 0 (0.02) | 0.07 | -0.02 (0.03) | 0.13 |
| D3 | E2 | 0.03 (0.03) | 0.13 | 0.01 (0.02) | 0.1 | 0.01 (0.02) | 0.07 | 0.07 (0.04) | 0.17 | 0 (0.02) | 0.06 | 0.08 (0.04) | 0.17 |
| D3 | E3 | 0 (0.01) | 0.05 | -0.01 (0.03) | 0.1 | 0.01 (0.02) | 0.09 | 0.02 (0.03) | 0.11 | 0.01 (0.02) | 0.09 | 0.01 (0.03) | 0.1 |
| D3 | E4 | -0.01 (0.02) | 0.08 | 0 (0.01) | 0.05 | 0.01 (0.02) | 0.09 | -0.03 (0.03) | 0.12 | 0.01 (0.03) | 0.1 | -0.03 (0.03) | 0.11 |
| D3 | E5 | 0.01 (0.01) | 0.06 | 0 (0.01) | 0.05 | -0.05 (0.04) | 0.15 | -0.01 (0.03) | 0.1 | -0.05 (0.04) | 0.15 | -0.02 (0.03) | 0.1 |
| D3 | E6 | 0 (0.01) | 0.04 | 0 (0.01) | 0.06 | 0.03 (0.04) | 0.15 | 0.02 (0.03) | 0.1 | 0.03 (0.03) | 0.14 | 0.02 (0.03) | 0.11 |
| D4 | D5 | 0.01 (0.02) | 0.06 | 0 (0.01) | 0.04 | 0.03 (0.03) | 0.1 | 0.04 (0.04) | 0.16 | 0.03 (0.03) | 0.11 | 0.04 (0.04) | 0.17 |
| D4 | D6 | 0.02 (0.03) | 0.11 | 0.01 (0.02) | 0.08 | 0 (0.02) | 0.09 | 0 (0.03) | 0.12 | 0.01 (0.02) | 0.09 | 0 (0.03) | 0.11 |
| D4 | D7 | 0.05 (0.04) | 0.16 | 0.03 (0.03) | 0.14 | -0.01 (0.02) | 0.09 | 0.04 (0.04) | 0.16 | -0.02 (0.02) | 0.09 | 0.04 (0.04) | 0.16 |
| D4 | E1 | 0.01 (0.02) | 0.07 | 0.02 (0.03) | 0.11 | 0 (0.02) | 0.08 | -0.08 (0.04) | 0.17 | 0 (0.02) | 0.09 | -0.08 (0.04) | 0.18 |
| D4 | E2 | 0.08 (0.04) | 0.18 | 0.07 (0.05) | 0.19 | 0.02 (0.02) | 0.09 | 0.03 (0.03) | 0.13 | 0.02 (0.02) | 0.09 | 0.03 (0.04) | 0.14 |
| D4 | E3 | 0.04 (0.04) | 0.15 | 0.04 (0.04) | 0.16 | 0 (0.02) | 0.09 | 0.01 (0.03) | 0.11 | 0 (0.02) | 0.09 | 0.01 (0.03) | 0.11 |
| D4 | E4 | 0.01 (0.02) | 0.1 | 0.04 (0.03) | 0.14 | 0.06 (0.05) | 0.18 | 0.01 (0.03) | 0.1 | 0.06 (0.04) | 0.18 | 0.02 (0.03) | 0.11 |
| D4 | E5 | 0.01 (0.01) | 0.06 | 0.01 (0.02) | 0.09 | 0.05 (0.04) | 0.18 | -0.01 (0.03) | 0.11 | 0.05 (0.04) | 0.17 | -0.01 (0.03) | 0.1 |
| D4 | E6 | 0.04 (0.03) | 0.14 | 0 (0.02) | 0.06 | 0.04 (0.04) | 0.16 | -0.01 (0.02) | 0.1 | 0.04 (0.04) | 0.16 | 0 (0.02) | 0.09 |
| D5 | D6 | 0.15 (0.05) | 0.18 | 0.23 (0.05) | 0.21 | 0.11 (0.04) | 0.16 | 0.08 (0.03) | 0.14 | 0.11 (0.04) | 0.16 | 0.07 (0.03) | 0.14 |
| D5 | D7 | 0.28 (0.04) | 0.17 | 0.24 (0.06) | 0.22 | 0.06 (0.04) | 0.14 | 0.03 (0.03) | 0.13 | 0.06 (0.04) | 0.14 | 0.03 (0.03) | 0.12 |
| D5 | E1 | 0.01 (0.01) | 0.06 | 0.02 (0.03) | 0.11 | 0 (0.02) | 0.07 | -0.04 (0.03) | 0.12 | 0 (0.02) | 0.08 | -0.03 (0.03) | 0.11 |
| D5 | E2 | 0.03 (0.03) | 0.13 | 0.04 (0.04) | 0.17 | 0.01 (0.02) | 0.09 | 0.02 (0.03) | 0.11 | 0.02 (0.02) | 0.09 | 0.03 (0.03) | 0.11 |
| D5 | E3 | 0.02 (0.03) | 0.11 | 0.01 (0.02) | 0.08 | 0.01 (0.02) | 0.09 | 0.06 (0.03) | 0.12 | 0.01 (0.02) | 0.09 | 0.05 (0.03) | 0.11 |
| D5 | E4 | 0 (0.01) | 0.05 | 0.01 (0.02) | 0.08 | 0 (0.02) | 0.1 | -0.01 (0.02) | 0.08 | -0.01 (0.03) | 0.11 | -0.01 (0.02) | 0.08 |
| D5 | E5 | 0.14 (0.04) | 0.18 | 0.08 (0.05) | 0.19 | 0.04 (0.04) | 0.16 | 0.05 (0.03) | 0.11 | 0.05 (0.04) | 0.16 | 0.05 (0.03) | 0.11 |
| D5 | E6 | 0 (0.02) | 0.06 | 0.01 (0.02) | 0.07 | 0.07 (0.05) | 0.18 | 0.02 (0.02) | 0.08 | 0.06 (0.04) | 0.18 | 0.02 (0.02) | 0.08 |
| D6 | D7 | 0.19 (0.04) | 0.17 | 0.27 (0.05) | 0.21 | 0.09 (0.03) | 0.14 | 0.05 (0.04) | 0.15 | 0.08 (0.03) | 0.14 | 0.05 (0.03) | 0.14 |
| D6 | E1 | 0 (0.01) | 0.05 | 0.02 (0.03) | 0.13 | 0 (0.02) | 0.07 | -0.05 (0.04) | 0.14 | 0.01 (0.02) | 0.09 | -0.04 (0.03) | 0.14 |
| D6 | E2 | 0.03 (0.03) | 0.13 | 0.01 (0.02) | 0.08 | 0.01 (0.02) | 0.08 | 0.04 (0.03) | 0.13 | 0.01 (0.02) | 0.09 | 0.05 (0.04) | 0.14 |
| D6 | E3 | 0.03 (0.03) | 0.14 | 0.07 (0.04) | 0.18 | 0.01 (0.02) | 0.09 | 0.01 (0.02) | 0.09 | 0.01 (0.02) | 0.1 | 0.01 (0.02) | 0.09 |
| D6 | E4 | 0.05 (0.04) | 0.15 | 0.06 (0.04) | 0.17 | 0 (0.02) | 0.09 | 0.04 (0.03) | 0.12 | 0 (0.02) | 0.1 | 0.04 (0.03) | 0.12 |
| D6 | E5 | 0.03 (0.03) | 0.12 | 0.04 (0.04) | 0.17 | -0.01 (0.03) | 0.13 | 0.04 (0.03) | 0.12 | -0.01 (0.03) | 0.12 | 0.04 (0.03) | 0.13 |
| D6 | E6 | 0.08 (0.04) | 0.16 | 0.04 (0.04) | 0.14 | 0.01 (0.03) | 0.11 | 0.01 (0.02) | 0.09 | 0.01 (0.03) | 0.1 | 0.02 (0.02) | 0.09 |
| D7 | E1 | 0.01 (0.02) | 0.06 | 0.07 (0.04) | 0.18 | 0.01 (0.02) | 0.1 | -0.02 (0.02) | 0.1 | 0.02 (0.03) | 0.11 | -0.01 (0.02) | 0.09 |
| D7 | E2 | 0.06 (0.04) | 0.16 | 0.06 (0.05) | 0.2 | 0.01 (0.02) | 0.08 | 0.02 (0.03) | 0.11 | 0.01 (0.02) | 0.08 | 0.03 (0.03) | 0.11 |
| D7 | E3 | 0 (0.01) | 0.05 | 0.01 (0.02) | 0.07 | 0.03 (0.03) | 0.13 | 0.03 (0.03) | 0.1 | 0.02 (0.03) | 0.11 | 0.03 (0.02) | 0.1 |
| D7 | E4 | 0 (0.01) | 0.04 | 0 (0.01) | 0.06 | 0 (0.02) | 0.1 | 0.02 (0.02) | 0.09 | -0.01 (0.03) | 0.12 | 0.02 (0.02) | 0.09 |
| D7 | E5 | 0.01 (0.02) | 0.09 | 0.07 (0.05) | 0.19 | 0.02 (0.03) | 0.13 | 0.02 (0.02) | 0.09 | 0.02 (0.03) | 0.13 | 0.02 (0.02) | 0.1 |
| D7 | E6 | 0 (0.01) | 0.04 | 0.01 (0.02) | 0.06 | 0 (0.03) | 0.12 | 0.02 (0.02) | 0.09 | 0 (0.03) | 0.11 | 0.03 (0.02) | 0.09 |
| E1 | E2 | 0.14 (0.05) | 0.18 | 0.16 (0.05) | 0.2 | 0 (0.01) | 0.06 | 0.07 (0.04) | 0.16 | 0 (0.01) | 0.06 | 0.08 (0.04) | 0.16 |
| E1 | E3 | 0 (0.01) | 0.06 | 0.04 (0.04) | 0.14 | 0 (0.02) | 0.08 | -0.01 (0.02) | 0.08 | 0 (0.02) | 0.08 | -0.01 (0.02) | 0.09 |
| E1 | E4 | 0.05 (0.04) | 0.15 | 0.01 (0.02) | 0.09 | 0 (0.02) | 0.1 | 0.04 (0.03) | 0.13 | 0 (0.02) | 0.1 | 0.03 (0.03) | 0.12 |
| E1 | E5 | 0.17 (0.04) | 0.18 | 0.1 (0.05) | 0.2 | 0.04 (0.03) | 0.14 | 0.01 (0.02) | 0.09 | 0.04 (0.04) | 0.14 | 0.02 (0.03) | 0.11 |
| E1 | E6 | 0.04 (0.04) | 0.15 | 0.1 (0.04) | 0.17 | 0.01 (0.03) | 0.12 | 0.01 (0.02) | 0.08 | 0.01 (0.03) | 0.12 | 0 (0.02) | 0.07 |
| E2 | E3 | 0.02 (0.03) | 0.12 | 0 (0.02) | 0.08 | 0.01 (0.02) | 0.1 | 0.01 (0.02) | 0.08 | 0.02 (0.03) | 0.1 | 0.01 (0.02) | 0.07 |
| E2 | E4 | 0.04 (0.04) | 0.14 | 0.01 (0.02) | 0.09 | 0.01 (0.03) | 0.1 | -0.01 (0.02) | 0.07 | 0.01 (0.03) | 0.11 | -0.01 (0.02) | 0.07 |
| E2 | E5 | 0.02 (0.03) | 0.1 | 0.11 (0.05) | 0.21 | 0.04 (0.04) | 0.15 | 0.01 (0.02) | 0.07 | 0.04 (0.04) | 0.14 | 0 (0.01) | 0.06 |
| E2 | E6 | 0.08 (0.04) | 0.18 | 0.1 (0.05) | 0.22 | 0.03 (0.04) | 0.15 | 0.02 (0.02) | 0.09 | 0.04 (0.04) | 0.16 | 0.01 (0.02) | 0.08 |
| E3 | E4 | 0.21 (0.04) | 0.17 | 0.23 (0.04) | 0.18 | 0.06 (0.04) | 0.15 | 0.03 (0.03) | 0.12 | 0.06 (0.04) | 0.16 | 0.03 (0.03) | 0.11 |
| E3 | E5 | 0 (0.01) | 0.05 | 0 (0.01) | 0.05 | 0.02 (0.03) | 0.12 | 0 (0.02) | 0.07 | 0.02 (0.03) | 0.12 | 0 (0.02) | 0.07 |
| E3 | E6 | 0.05 (0.04) | 0.15 | 0.01 (0.02) | 0.09 | 0.03 (0.04) | 0.14 | 0.01 (0.02) | 0.07 | 0.03 (0.04) | 0.14 | 0.01 (0.02) | 0.08 |
| E4 | E5 | 0.06 (0.04) | 0.16 | 0.01 (0.02) | 0.1 | 0.01 (0.03) | 0.11 | -0.03 (0.03) | 0.14 | 0.01 (0.03) | 0.1 | -0.03 (0.03) | 0.13 |
| E4 | E6 | 0.05 (0.04) | 0.16 | 0.17 (0.04) | 0.17 | 0.02 (0.03) | 0.13 | 0.04 (0.03) | 0.13 | 0.02 (0.03) | 0.12 | 0.04 (0.03) | 0.14 |
| E5 | E6 | 0.14 (0.05) | 0.18 | 0.2 (0.04) | 0.17 | 0.01 (0.03) | 0.12 | 0.07 (0.04) | 0.14 | 0.02 (0.03) | 0.13 | 0.07 (0.04) | 0.15 |

Note. N1 = node 1; N2 = node 2.

**Table S3.**

*Standardized symptom centrality values in cross-sectional and cross-lagged panel network models*

| Note | EI_Pre | EI_post | OutEI_CLPN | InEI_CLPN | OutEI_CLPN (with covariates) | InEI_CLPN (with covariates) |
| --- | --- | --- | --- | --- | --- | --- |
| B1 | 0.87 | **1.56** | -0.06 | 0.62 | 0.10 | 0.77 |
| B2 | -0.03 | -0.26 | -1.49 | **1.35** | -1.39 | **1.19** |
| B3 | 0.25 | **1.11** | 0.10 | -0.07 | 0.12 | 0.00 |
| B4 | **1.31** | 0.81 | -1.52 | 0.91 | -1.00 | **1.00** |
| B5 | **1.13** | **1.06** | **1.44** | 0.61 | **1.38** | 0.73 |
| C1 | -0.92 | 0.30 | -1.95 | 0.99 | -2.14 | 0.48 |
| C2 | -0.32 | -0.72 | -0.33 | **1.40** | -0.40 | **1.47** |
| D1 | -2.07 | -1.79 | 0.29 | -1.00 | 0.38 | -0.86 |
| D2 | **1.05** | 0.93 | 0.06 | 0.42 | -0.06 | 0.65 |
| D3 | -0.22 | -0.63 | -0.43 | 0.05 | -0.36 | 0.35 |
| D4 | 0.93 | 0.21 | **1.51** | -0.58 | **1.45** | -0.42 |
| D5 | -0.52 | -0.29 | **1.60** | -0.37 | **1.83** | -0.59 |
| D6 | **1.95** | **1.11** | 0.30 | **1.08** | 0.39 | **1.21** |
| D7 | 0.51 | **1.26** | 0.29 | 0.68 | 0.01 | 0.76 |
| E1 | -1.37 | -1.50 | -1.35 | -1.44 | -1.43 | -1.04 |
| E2 | -1.08 | -1.03 | 0.91 | -1.90 | **1.06** | -1.52 |
| E3 | -0.37 | -0.15 | 0.42 | -0.92 | 0.06 | -1.24 |
| E4 | -0.34 | -1.19 | -0.12 | -1.13 | -0.12 | -1.07 |
| E5 | -0.48 | -0.86 | -0.21 | -1.16 | -0.35 | -1.78 |
| E6 | -0.28 | 0.07 | 0.53 | 0.46 | 0.46 | -0.07 |

Note. Centrality values with a *z*-score > 1.0 are bolded. EI = expected influence; Pre = pre-treatment network; post = post treatment network; outEI = outdegree expected influence; inEI = indegree expected influence; CLPN = cross-lagged panel network.

**Table S4**

*Subgroup network analyses by demographics at pre- and post-treatment*

| Subgroup | Core symptoms at Pre-treatment | Core symptoms at Post-treatment | Within-group Paired NCT | Between-group NCT  (pre-treatment) | Between-group NCT  (post-treatment) |
| --- | --- | --- | --- | --- | --- |
| **Age** |  |  |  | Structure (*p =*.075)  Strength (*p =*.501) | Structure (*p =*.076)  Strength adolescents > children (*p =*.007) |
| Children | "B1" "B5" "D2" "D4" "D6" | "B1" "B3" "D2" "D7" | Structure (*p =*.294)  Strength (*p =*.331) |  |  |
| Adolescents | "B4" "D6" | "B1" "B5" "D6" | Structure (*p =*.342)  Strength post > pre (***p =*.002)** |  |  |
| **Sex** |  |  |  | Structure (*p =*.780)  Strength (*p =*.674) | Structure (*p =*.602)  Strength (*p =*.967) |
| Boy | "B5" "D2" "D6" | B1" "B3" "B5" "D7" | Structure (*p =*.460)  Strength (*p =*.141) |  |  |
| Girl | "B1" "B4" "D6" | "B1" "B4" "D2" "D6" "D7" | Structure (*p =*.998)  Strength post > pre (*p =*.020) |  |  |
| **Race** |  |  |  | Structure (*p =*.399)  Strength (*p =*.708) | Structure (*p =*.611)  Strength (*p =*.708) |
| White | "B1" "B4" "B5" "D2" "D6" | "B1" "B4" "B5" "D2" "D7" | Structure (*p =*.426)  Strength post > pre (***p =*.001**) |  |  |
| BIPOC | "B3" "B4" "B5" "D4" "D6" | "B1" "B3" "D6" | Structure (*p =*.221)  Strength post > pre (*p =*.010) |  |  |
| **Index Trauma** |  |  |  | Structure (*p =*.157)  Strength (*p =*.420) | Structure (*p =*.436)  Strength (*p =*.585) |
| Child Maltreatment | "B1" "B4" "B5" "D6" | "B1" "B3" "B4" "D2" "D6" "D7" | Structure (*p =*.788)  Strength post > pre (*p =*.021) |  |  |
| Other Index Trauma | "B4" "B5" "D2" "D4" "D6" | "B1" "B5" "D7" | Structure (*p =*.470)  Strength (*p =*.080) |  |  |

Note. NCT: network comparison test. BIPOC: Black, Indigenous, and People of Color. *p*-values that are less than the Bonferroni adjusted critical value = .003 for multiple comparisons are in bold. It is noteworthy that subgroup analyses were generally adequately powered based on the *netSimulator* function from the “bootnet” package. Some paired NCTs may have been underpowered, given recommendations of approximately 500 participants for networks with 20 nodes and 0.1 density (van Borkulo et al., 2023).

**Table S5**

*Autoregressive effects from the Cross-Lagged Panel Network (CLPN) models*

| Symptom | CLPN | CLPN  (with covariate) |
| --- | --- | --- |
| B1 | 0.10 | 0.10 |
| B2 | 0.12 | 0.11 |
| B3 | 0.09 | 0.08 |
| B4 | 0.04 | 0.03 |
| B5 | 0.14 | 0.14 |
| C1 | 0.00 | 0.00 |
| C2 | 0.09 | 0.09 |
| D1 | 0.19 | 0.18 |
| D2 | 0.23 | 0.23 |
| D3 | 0.15 | 0.14 |
| D4 | 0.24 | 0.22 |
| D5 | 0.18 | 0.19 |
| D6 | 0.11 | 0.10 |
| D7 | 0.15 | 0.15 |
| E1 | 0.13 | 0.14 |
| E2 | 0.19 | 0.20 |
| E3 | 0.18 | 0.17 |
| E4 | 0.16 | 0.16 |
| E5 | 0.31 | 0.30 |
| E6 | 0.19 | 0.20 |

Note. CLPN: Cross-lagged Panel Network

**Figure S1.** *Bootstrap 95% confidence intervals for estimated edge weights for the symptom network at (a) pre-treatment (b) post-treatment (c) CLPN without covariate and (d) CLPN with covariate*

| A Pre-treatment Cross-Sectional Edge Weights | B Post-treatment Cross-Sectional Edge Weights |
| --- | --- |
| 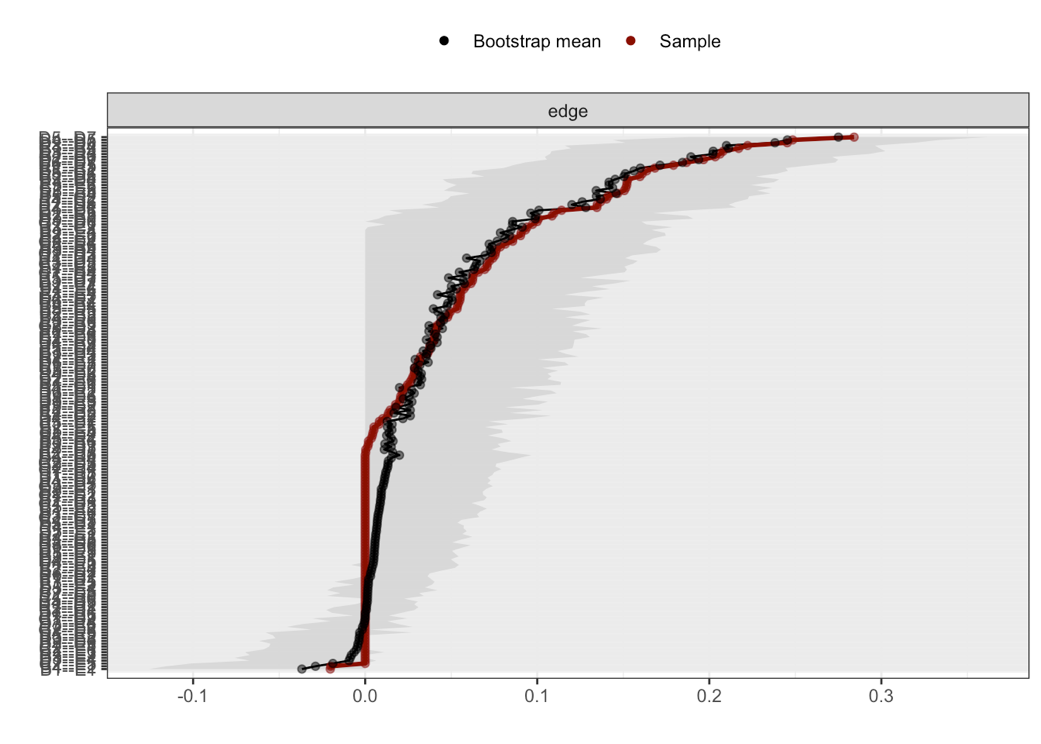 | 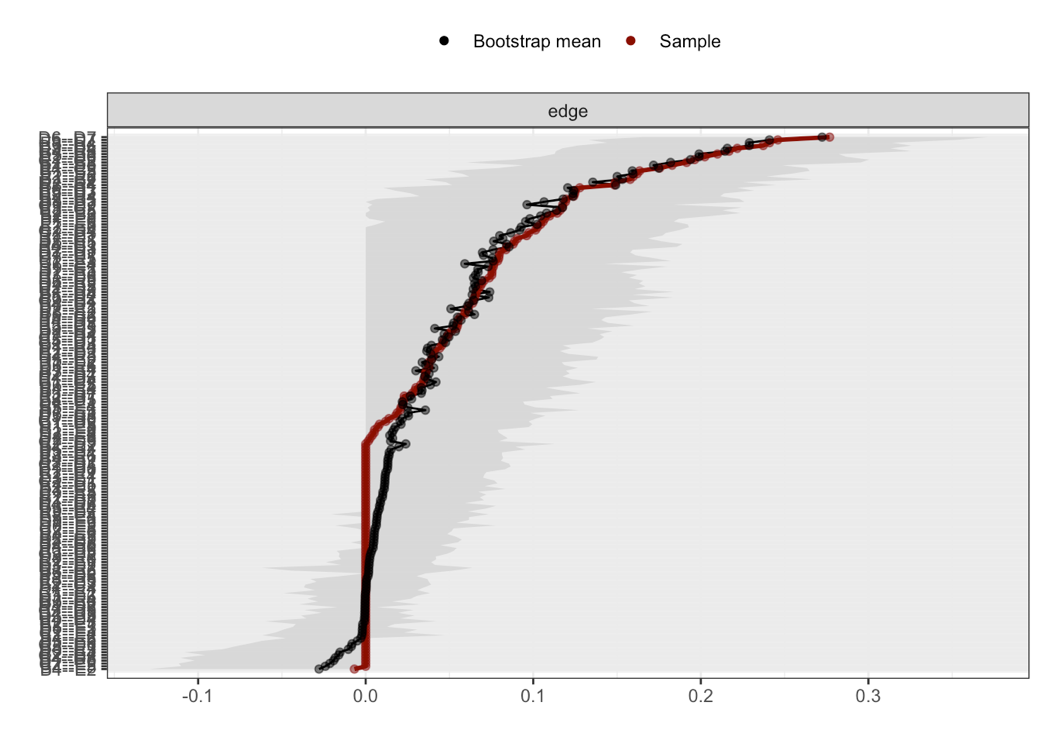 |
| C Unadjusted CLPN Edge Weights | D CLPN Edge Weights Adjusted for Covariates |
| 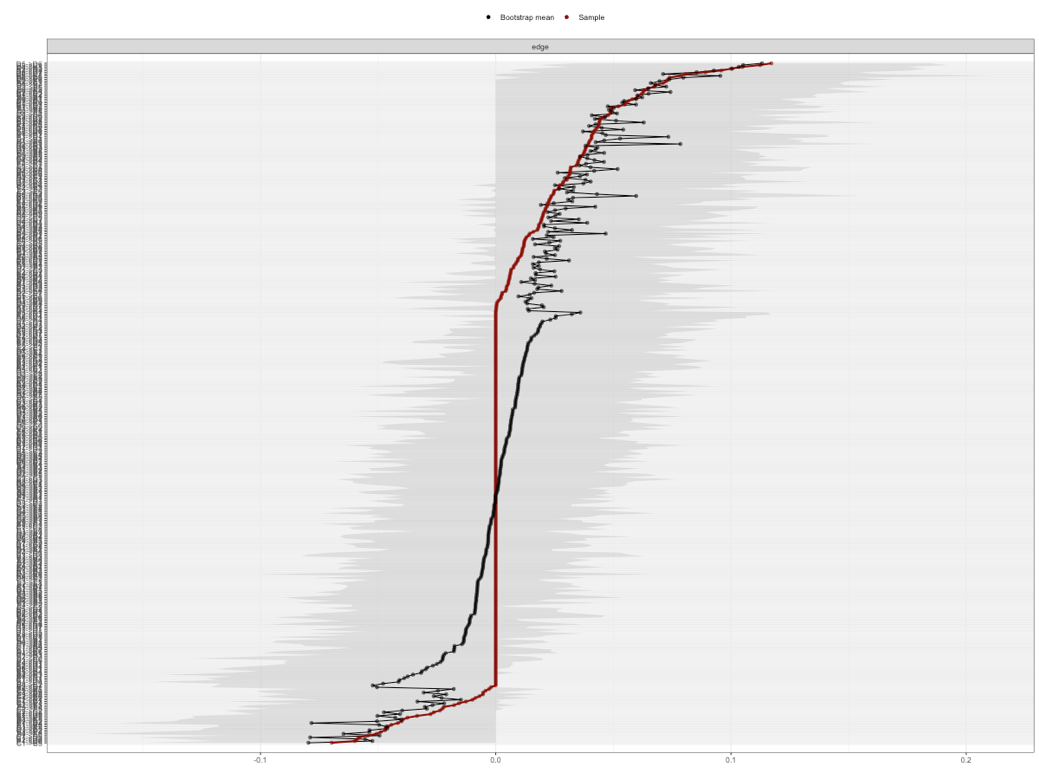 | 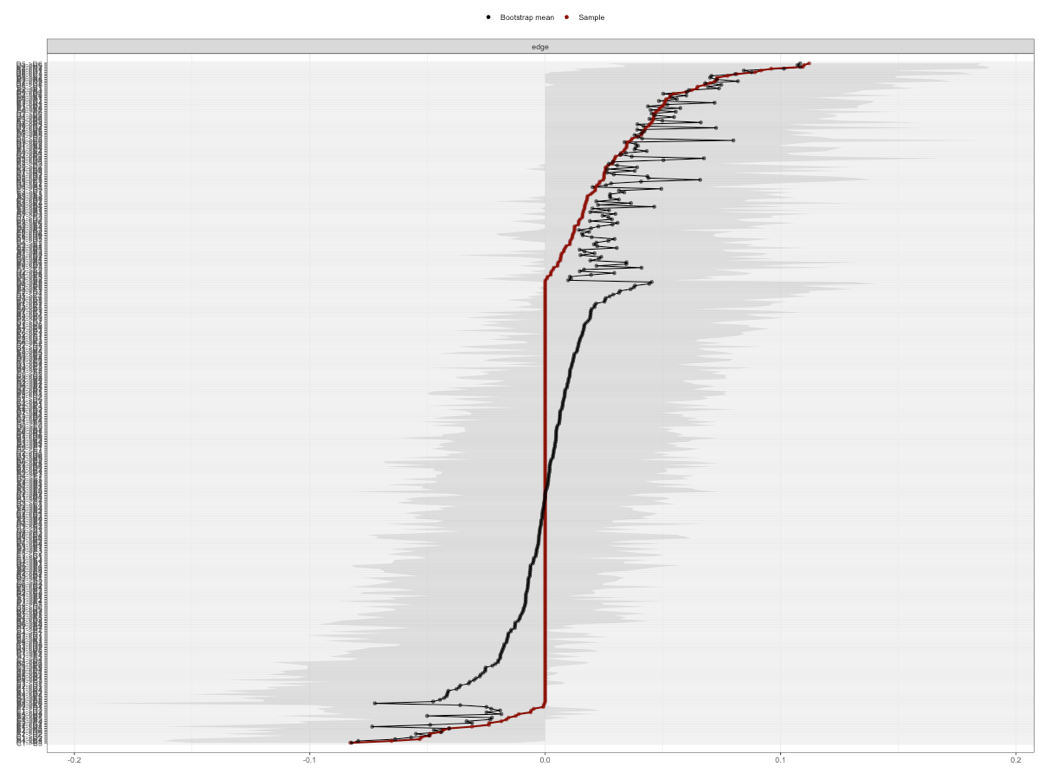 |

Note. CLPN: cross-lagged panel network. The red line represents the original sample estimate of edge weights. Each horizontal gray bar represents the 95% CI for a given edge from 1,000 bootstrap resamples and the black line shows the mean edge weight across bootstrap samples. Edge weights are sorted along the y-axis by magnitude.

**Figure S2.** *Bootstrap edge weights difference test between non-zero estimated edge-weights in the network of PTSD symptoms at (a) pre-treatment (b) post-treatment (c) CLPN without covariate and (d) CLPN with covariate*

| a | b |
| --- | --- |
| 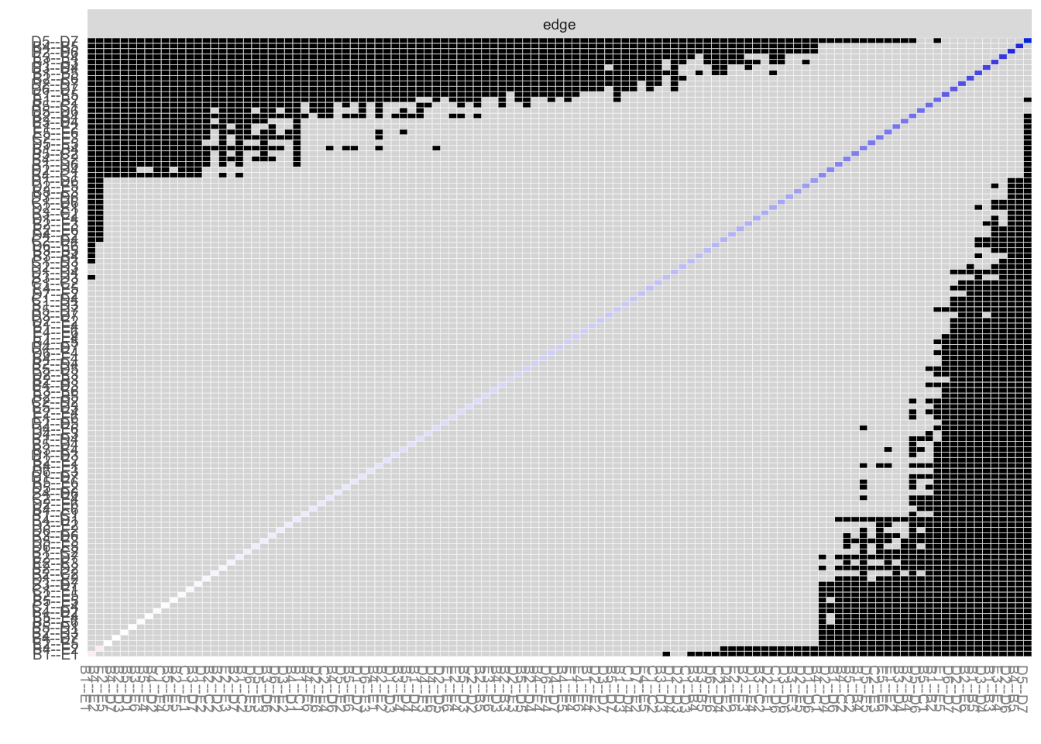 | 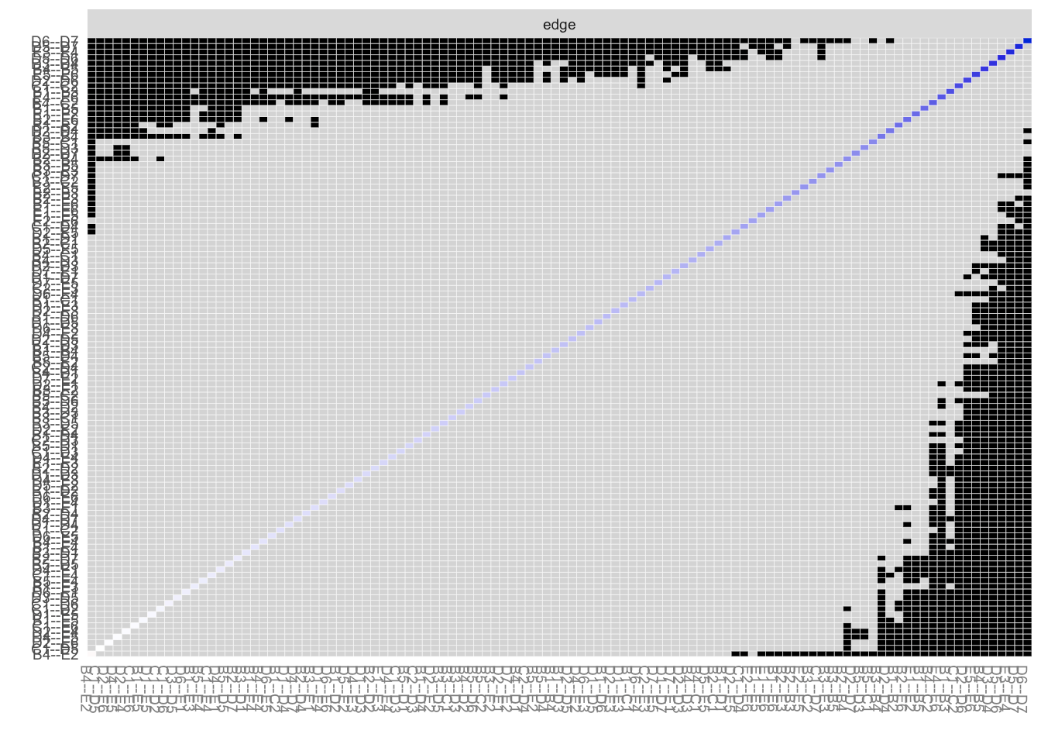 |
| c | d |
| 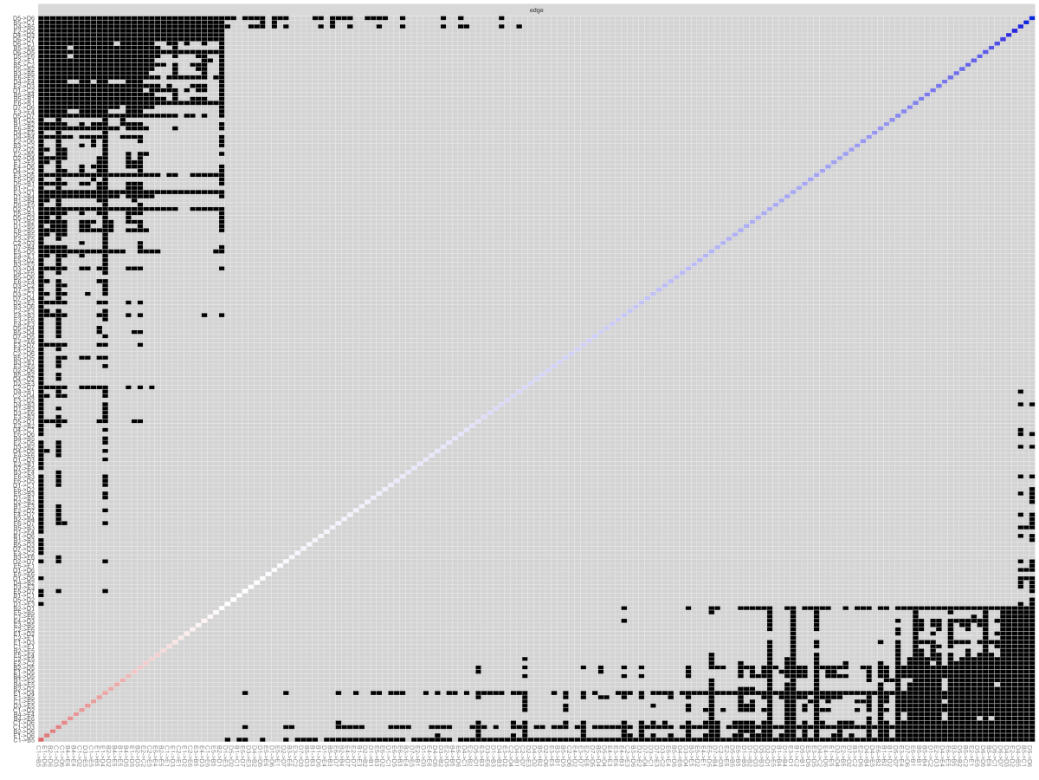 | 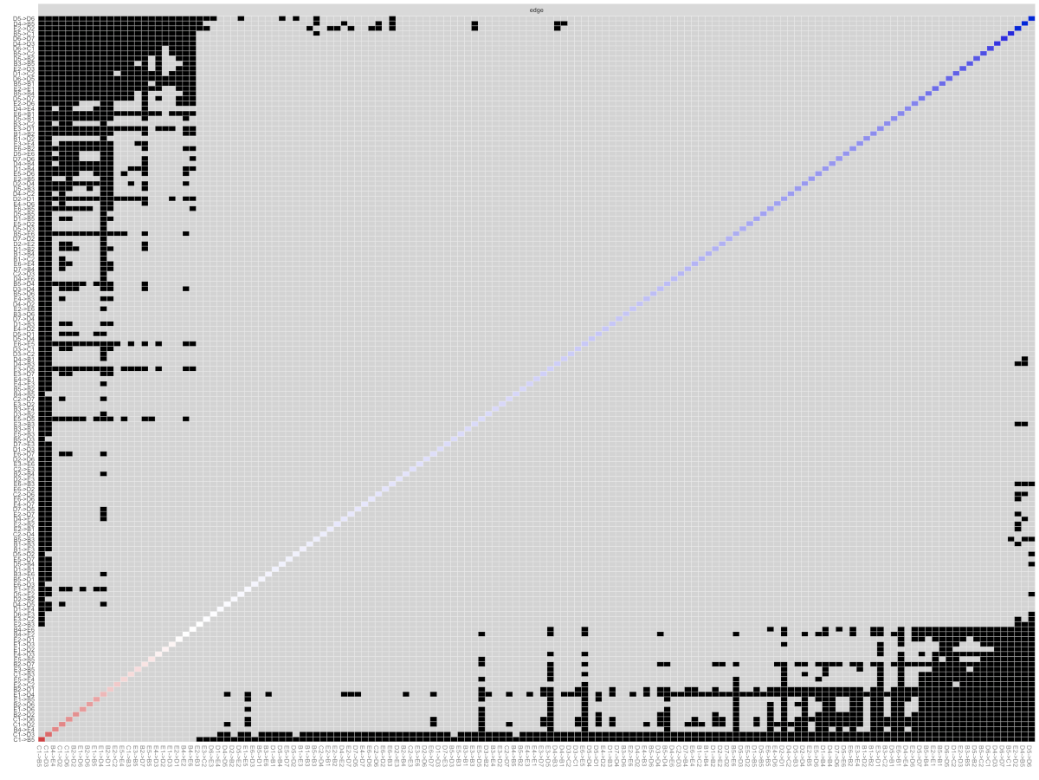 |

*Note.* Bootstrapped difference tests (α = .05) for pairwise comparisons between edge weights in the baseline PTSD symptom network. Each cell represents the comparison between two edge weights. Black boxes indicate a statistically significant difference between the edge weights (*p* < .05), whereas gray boxes indicate non-significant differences. The diagonal boxes the magnitude of the edge weight, with color (red = negative; white = 0; blue = positive) indicating the direction and strength (darker color represents stronger edge) of edge weight.

**Figure S3.**

*The average correlation between bootstrap centrality measures of networks sampled with case-dropping and the symptom network at (a) pre-treatment cross-sectional (b) post-treatment cross-sectional (c) CLPN without covariate and (d) CLPN with covariate*

| a | b |
| --- | --- |
| 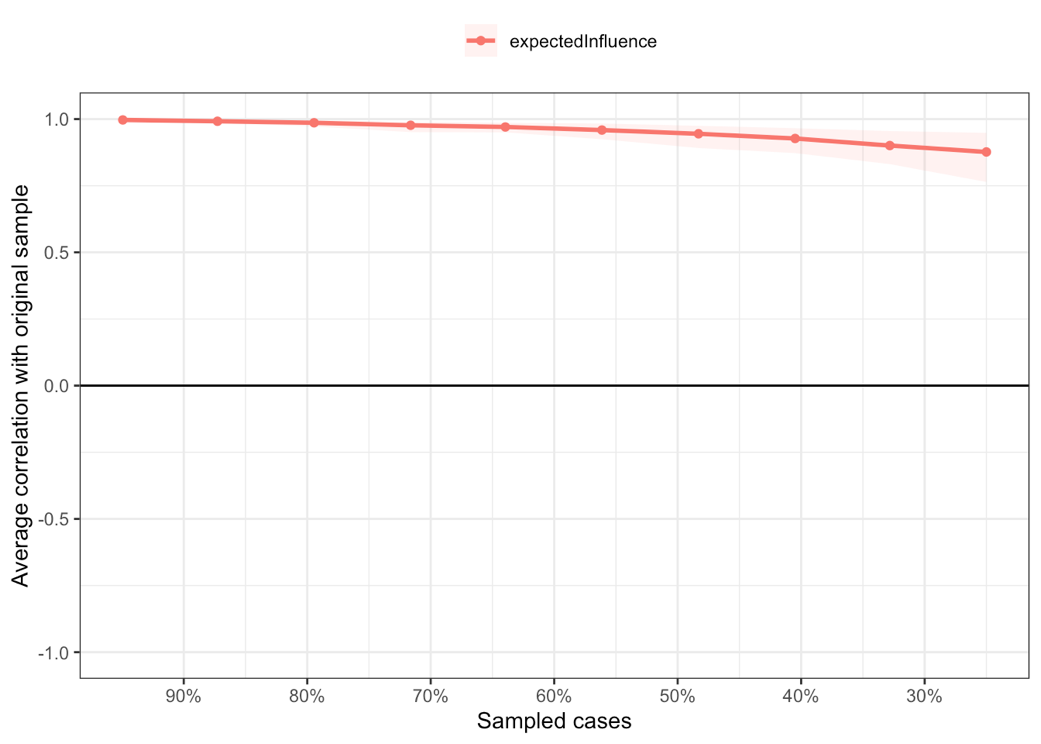 | 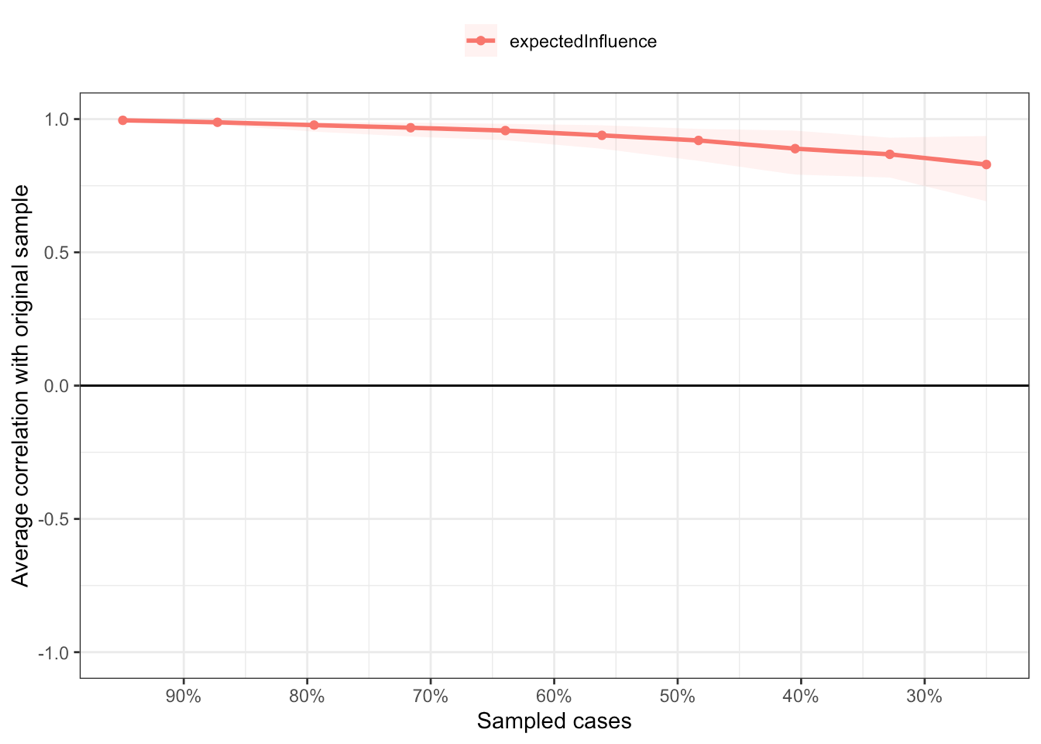 |
| c | d |
| 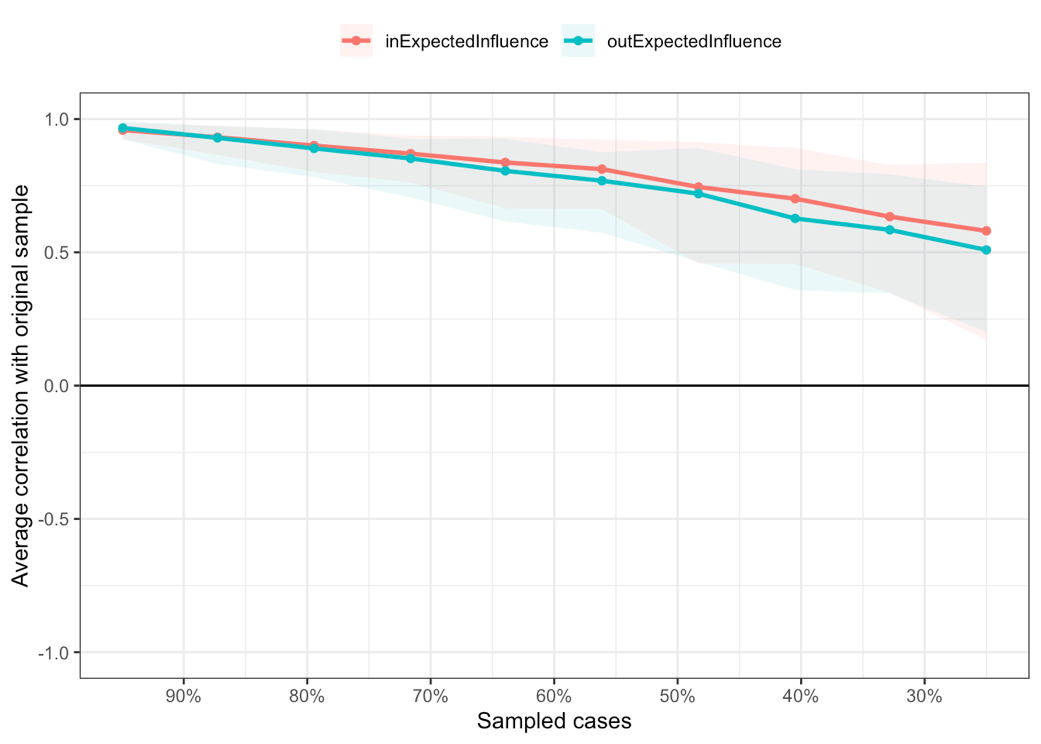 | 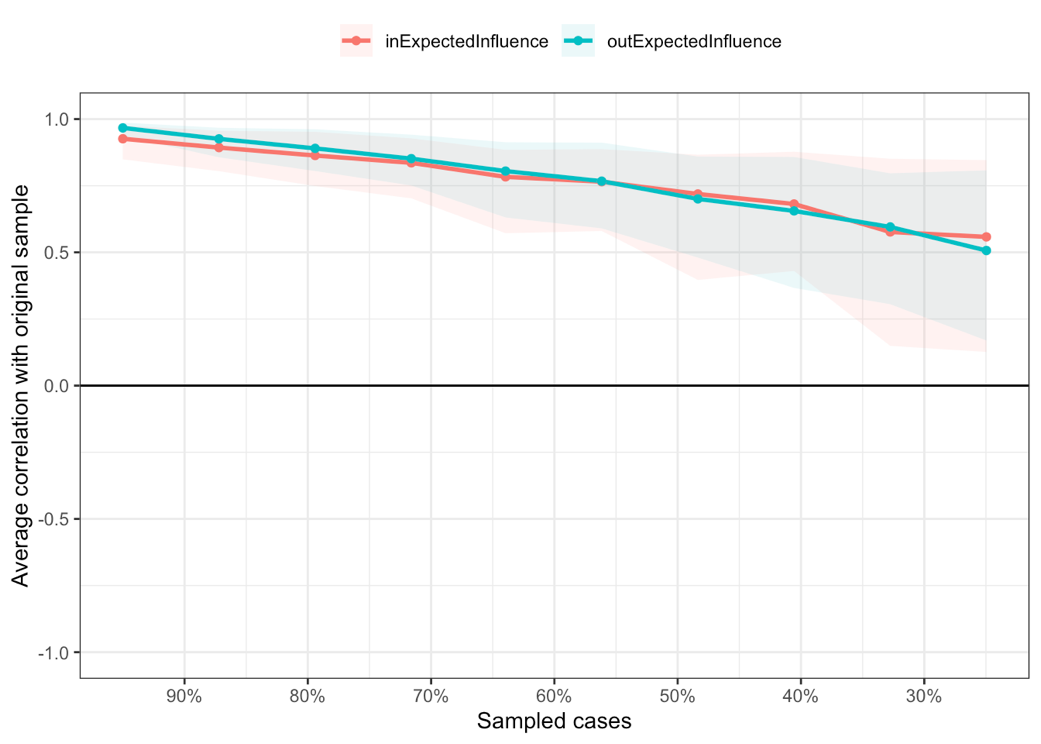 |

Note. The horizontal black line represents 0 correlation. Sampled cases represents the proportion of cases in the sample upon with the estimate of the centrality measure was based.

**Figure S4.** *Bootstrap node expected influence difference test between nodes of the PTSD symptom structure at (a), post-treatment (b), CLPN without covariate (c: out-EI, d: in-EI), and CLPN with covariate (e: out-EI, f: in-EI).*

| 1. pre-treatment | b |
| --- | --- |
| 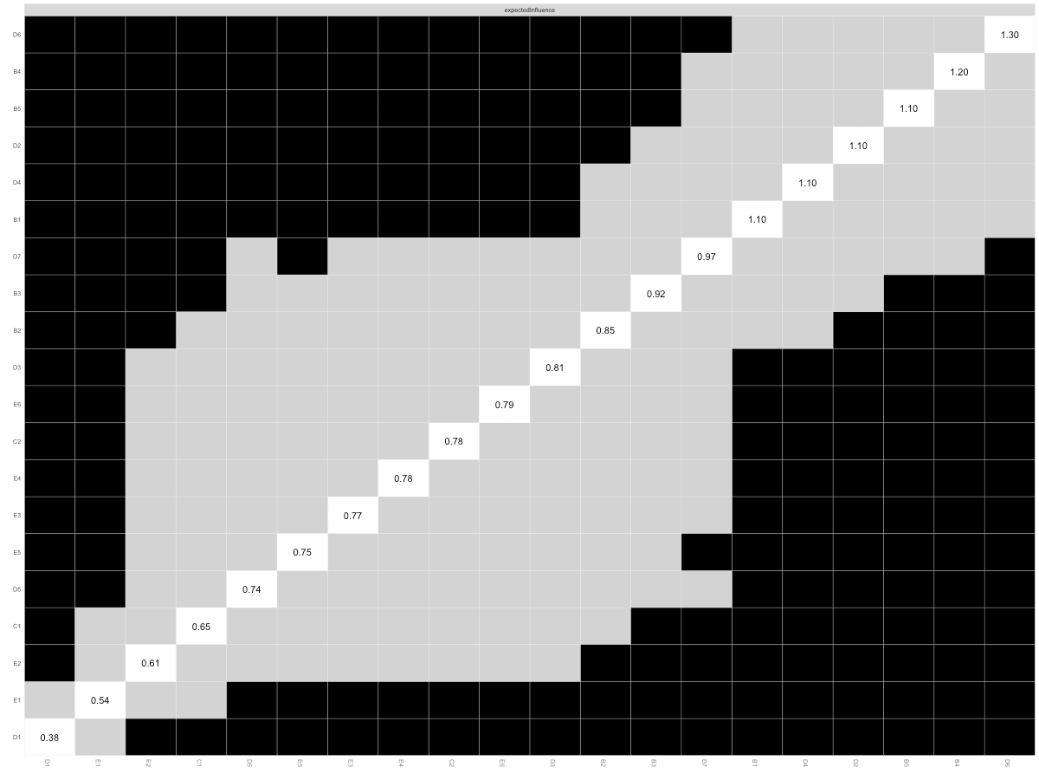 | 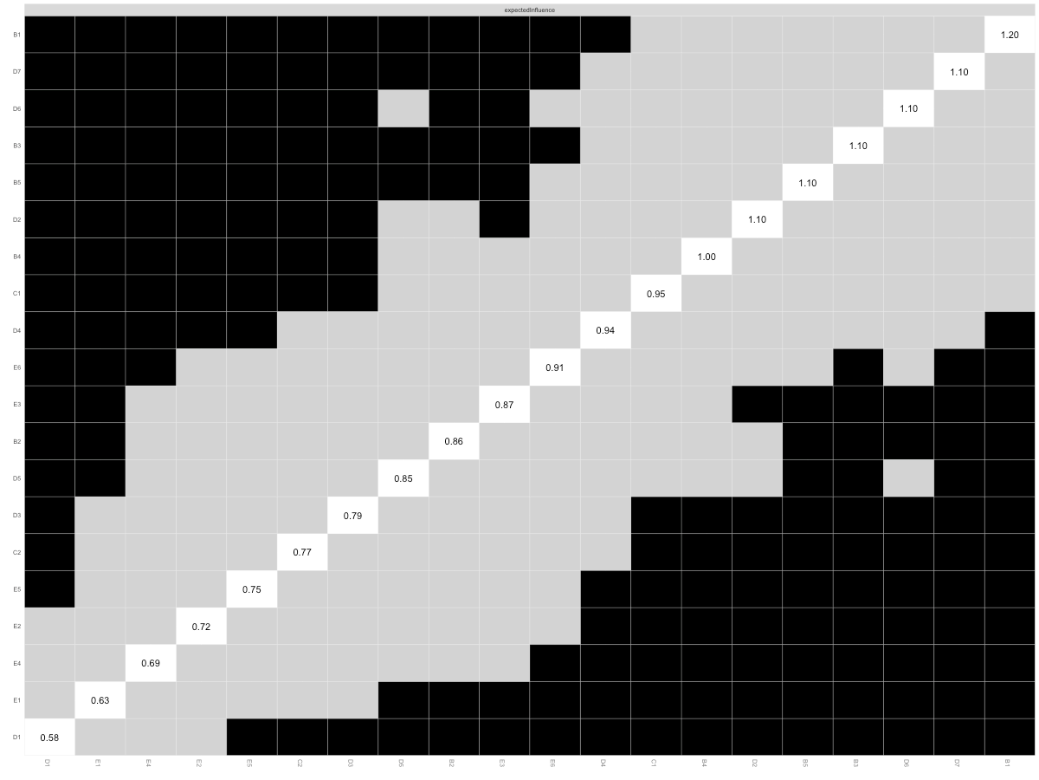 |
| c | d |
| 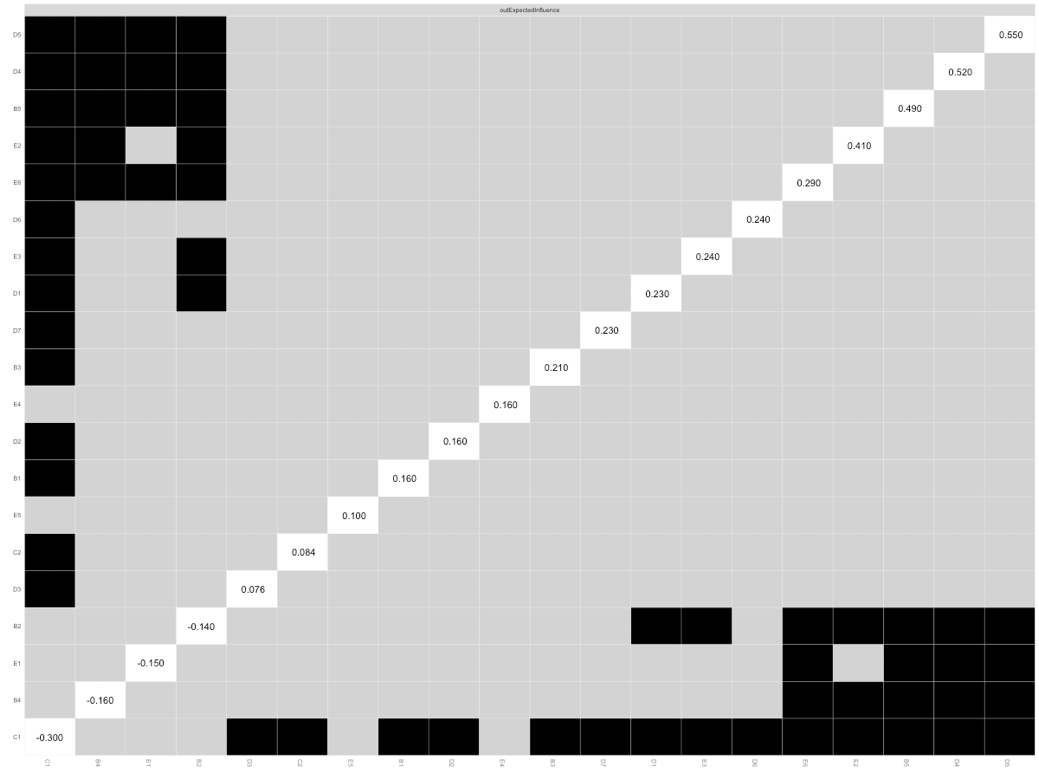 | 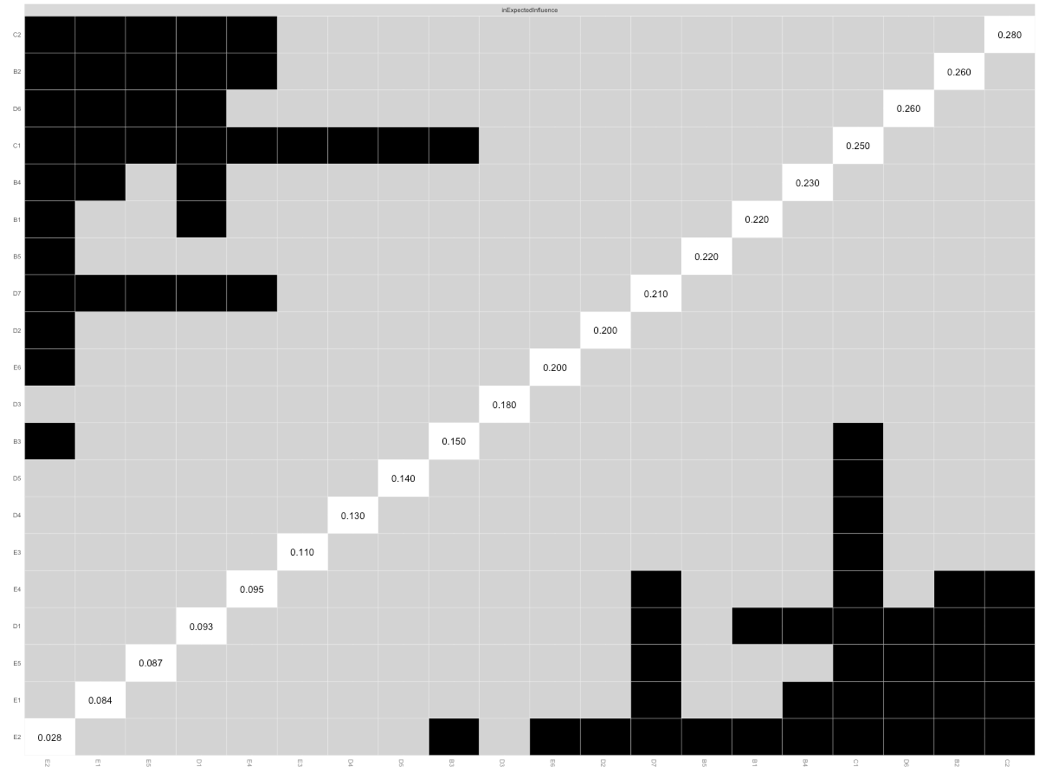 |
| E | f |
| 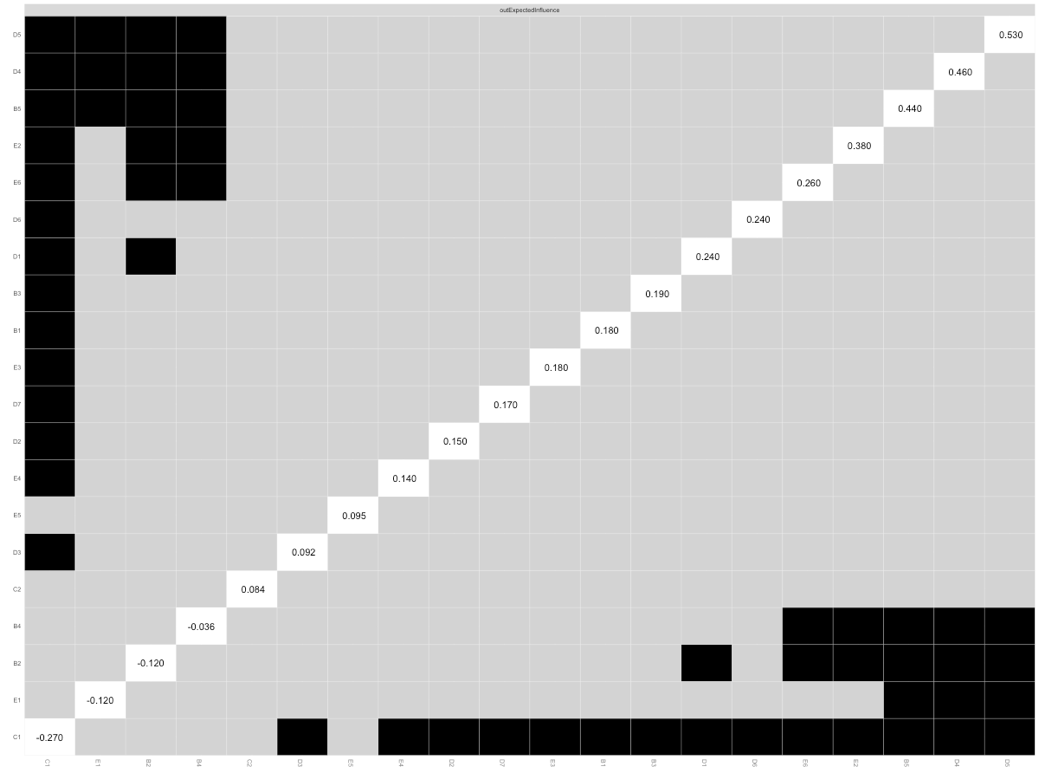 | 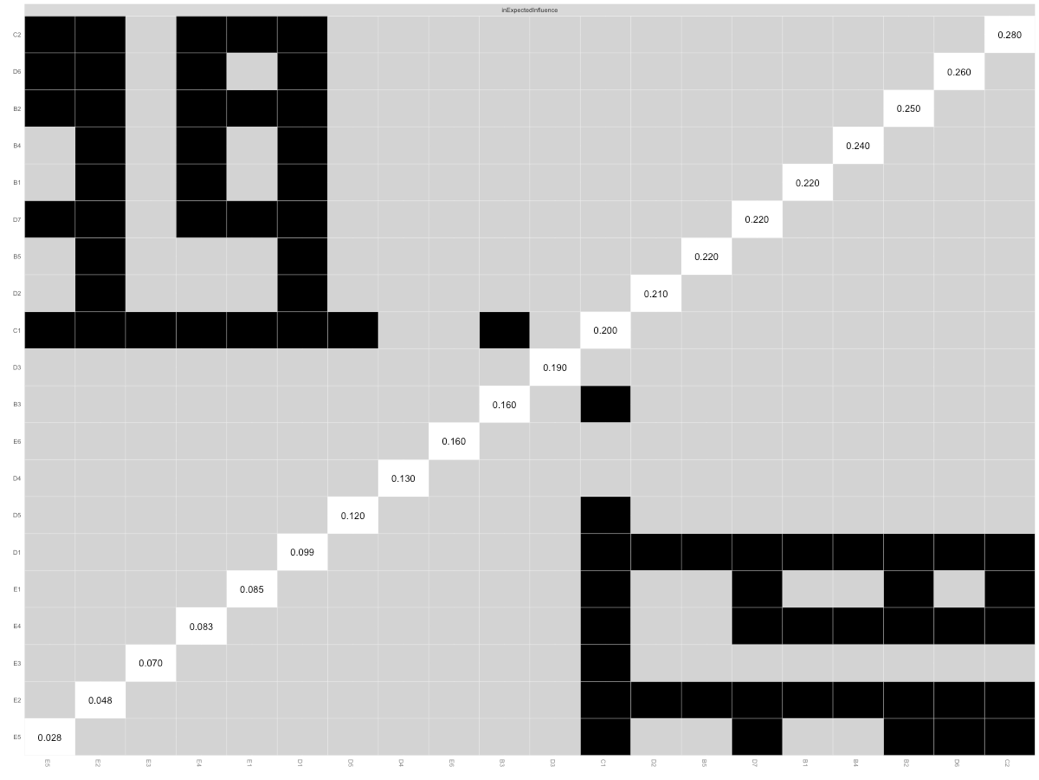 |

*Note.* Bootstrapped difference test (α = .05) comparing centrality indices for PTSD symptoms in the network. Both X-axis and Y-axis are 20 PTSD symptoms and the diagonal value displayed the specific nodes centrality index value. Each white diagonal box shows the centrality value of a node (symptom) based on the original sample. Black cells indicate statistically significant differences in centrality between two nodes (p < .05); gray cells indicate non-significant differences. These comparisons help identify which symptoms are significantly more central (i.e., more strongly connected) than others.

**Figure S5** *Adjusted CLPN PTSD symptom network at post-treatment and centrality indexes.*

| 1. Cross‑lagged effects (all estimated paths) | 1. Cross‑lagged effects with absolute value > 0.10 |
| --- | --- |
| 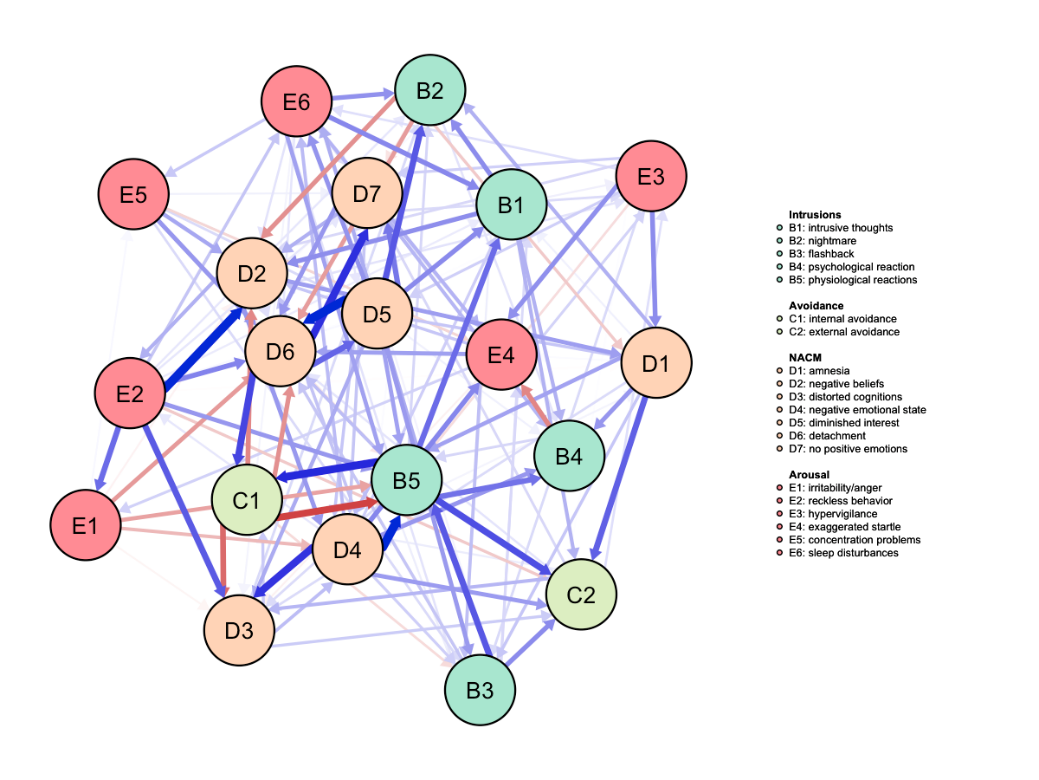 | 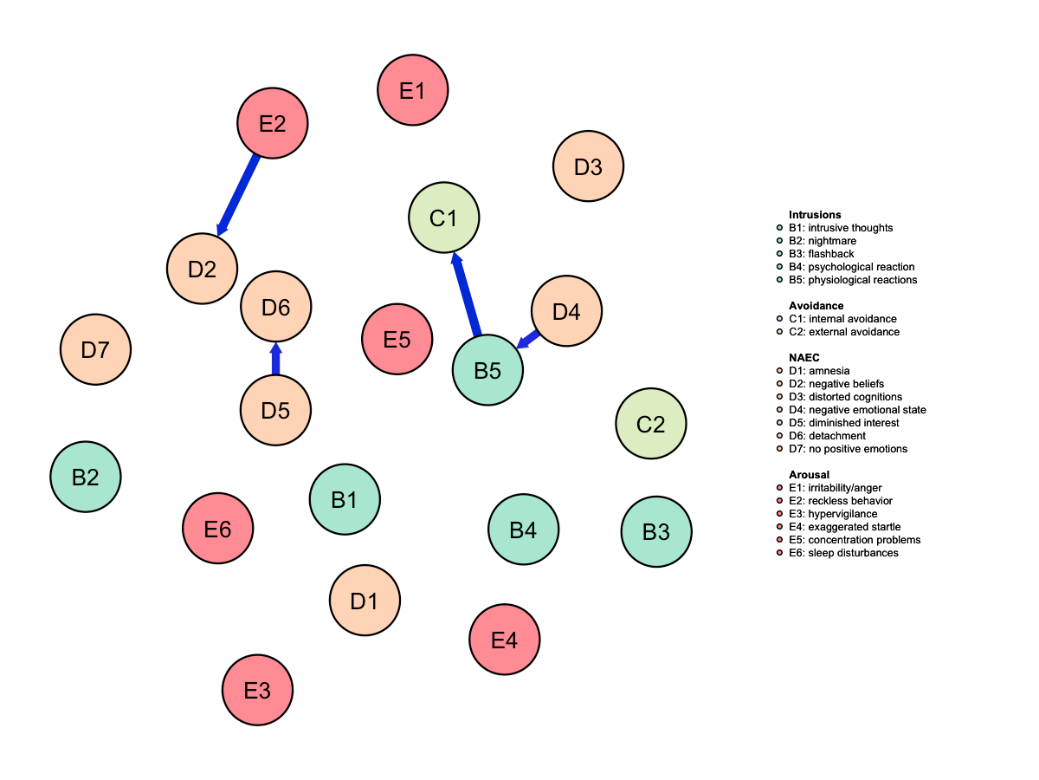 |
| 1. Centrality index (standardized z‑score) | 1. Autoregressive effects (within‑node stability) |
| 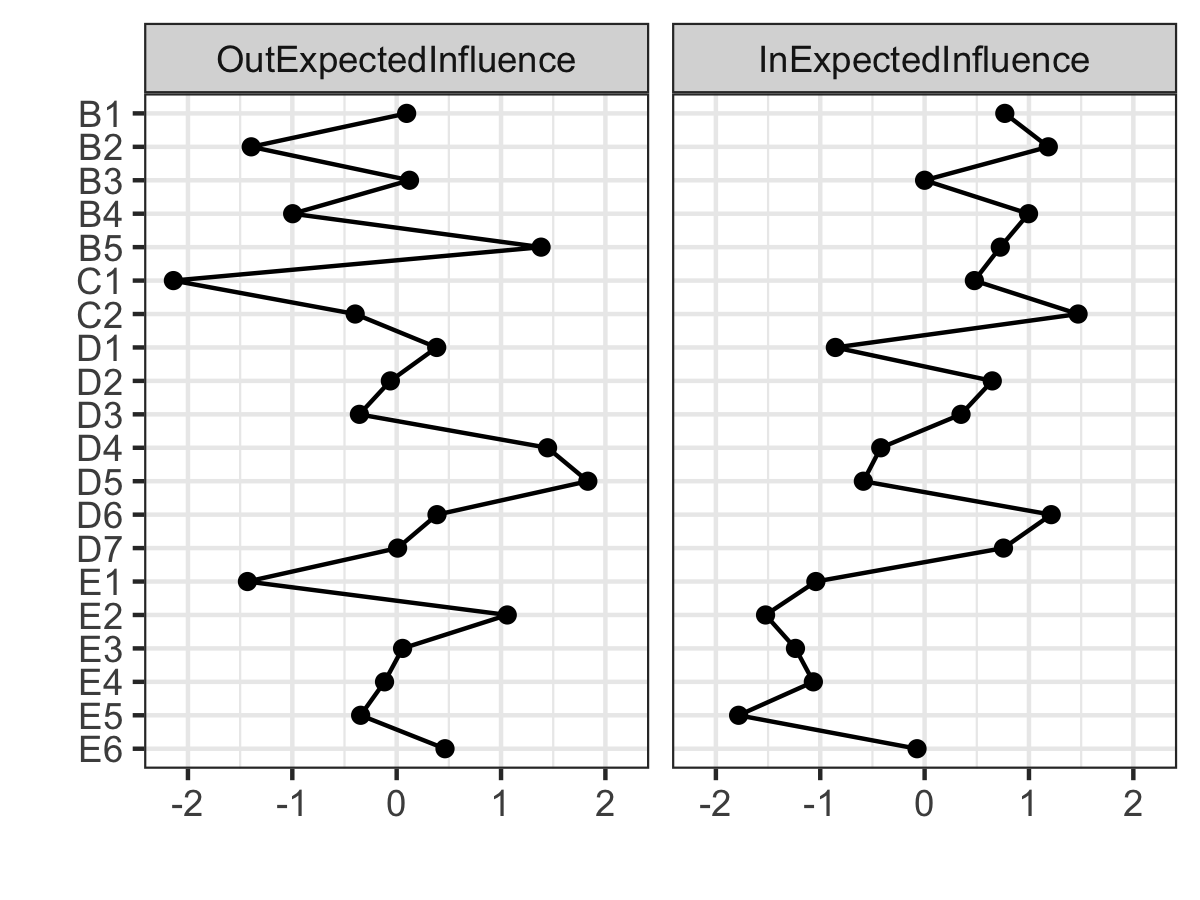 | 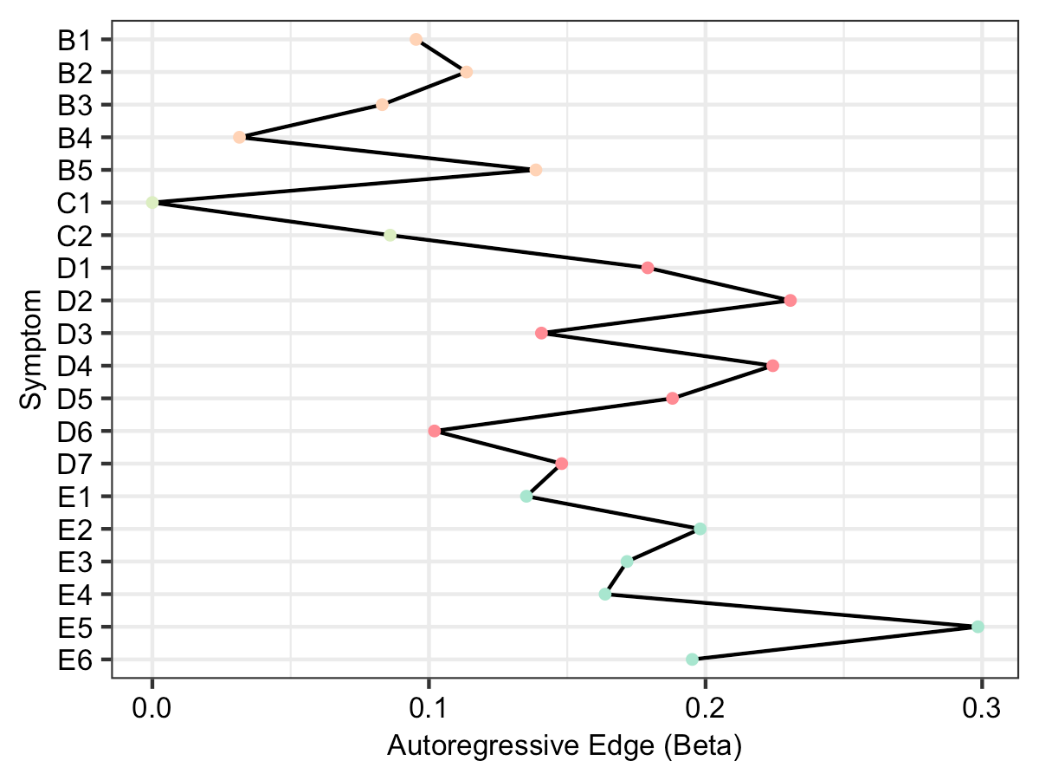 |

*Note*. The CLPN was adjusted for baseline age, sex, race, and child maltreatment status. NACM = Negative Alterations in Cognitions and Mood.
